# Factors influencing healthcare access among older adults in Southeast Asia: a scoping review guided by the levesque framework

**DOI:** 10.1186/s12913-025-13838-8

**Published:** 2025-12-07

**Authors:** Abhijith A. Kumar, Anu Mohan, K. Rakshitha, Priyobrat Rajkhowa, Teddy Andrews Jaihind Jothikaran, Lena Ashok, Asha Kamath

**Affiliations:** 1 Department of Applied Statistics and Data Science, Prasanna School of Public Health, Manipal Academy of Higher Education, Manipal, India; 2 Department of Social and Health Innovation, Prasanna School of Public Health, Manipal Academy of Higher Education, Manipal, India; 3 Department of Global Public Health Policy and Governance, Prasanna School of Public Health, Manipal Academy of Higher Education (MAHE), Manipal, Karnataka 576104 India

**Keywords:** Older adults, Healthcare utilization, WHO-Southeast Asia, Levesque framework, Health care access

## Abstract

**Background:**

Access to healthcare among the aging population in the WHO Southeast Asia (WHO-SEARO) encounters several challenges, resulting in unmet healthcare needs in this region. These challenges are associated with adverse health outcomes, including the growing prevalence of multimorbidity (also referred to as multiple long-term conditions), and heightened economic and systemic burdens on healthcare infrastructures.

**Method:**

This study aimed to identify factors influencing healthcare access among older individuals in the WHO-SEARO, adopting a Scoping review methodology based on the Arksey and O’Malley Framework (2005) and the Levesque conceptual framework for access to healthcare.

**Results:**

Multiple databases, including Scopus, PubMed, Web of Science, and CINAHL, were screened, resulting in the identification and analysis of 39 articles. The findings highlight significant progress in healthcare access among older adults in WHO-SEARO. However, inadequate health information, heightened economic dependency and treatment cost, limited accessibility and social support, often impede optimal healthcare utilization among older adults.

**Conclusion:**

Sustained and improved healthcare access for older adults demands prioritising proactive policies, targeted aged care incentives and, decentralized programs targeting older adults across different socio-demographic milieus.

**Supplementary Information:**

The online version contains supplementary material available at 10.1186/s12913-025-13838-8.

## Introduction

The South-East Asia Region has witnessed notable advancement in healthcare utilization over the past decade, driven by modern medical technologies, pharmaceutical innovations, and improvements in healthcare infrastructure [1, 2]. The World Health Organization South-East Asia Regional Office (WHO-SEARO), home to approximately 1.94 billion people, plays a central role in supporting global health efforts and Sustainable Development Goals (SDGs) in this region [3, 4]. Importantly, this region is undergoing a significant demographic transition and is projected to become the largest global hub for older adults by 2050 [5, 6].

Despite these advances, challenges remain in achieving equitable healthcare access for older adults [7, 8]. Over half of the population in the region still lacks access to essential healthcare services, and approximately 100 million people are pushed into extreme poverty each year due to out-of-pocket medical expenses, which contributes to increased vulnerability and reduced quality of life [9, 10]. These disparities are further exacerbated by the growing burden of multiple long-term conditions (MLTCs), which significantly impact the healthcare needs and utilization patterns of older adults [11, 12].

Although convergence in health outcomes has been observed among younger populations, disparities persist among working-age and older adults, indicating the need for targeted strategies to improve healthcare access and quality for ageing populations [13, 14]. While several efforts have been made to systematically map healthcare access [7], existing literature lacks sufficient evidence on both systemic and non-systemic enablers and barriers to healthcare access among older adults in the WHO South-East Asia Region.

Guided by Levesque’s conceptual framework for healthcare access [15], this scoping review seeks to identify the barriers and facilitators influencing healthcare access among older adults in WHO-SEARO. This framework conceptualizes access as the opportunity to recognize healthcare needs, seek, reach, obtain, and ultimately benefit from health services. It adopts a multidimensional approach, integrating system-level dimensions such as approachability, acceptability, availability, affordability, and appropriateness with individual abilities to perceive, seek, reach, pay for, and engage with healthcare. The framework’s emphasis on the appropriateness dimension stands out for its focus on the quality of communication, trust, and respect in healthcare interactions, which are crucial aspects of patient-centered and respectful care, especially for older adults. Understanding these interlinked factors is essential for informing policies and interventions aimed at improving equitable healthcare access in this rapidly ageing region. The unique contribution of this review lies in its ability to inform targeted, evidence-based policy recommendations. These recommendations aim to guide policymakers in designing strategies that improve equitable healthcare access, enhance service acceptability and appropriateness, and promote effective patient-provider communication specifically for ageing populations in WHO-SEARO. Ultimately, this review supports the development of policies that address complex barriers and facilitators in healthcare utilization, thereby improving health outcomes and quality of life for older adults in the region.

## Methods

The scoping review adopted the Arksey and O’Malley framework (2005) to conduct this study [16]. To ensure adequate coverage of necessary elements in reporting, the ‘Preferred Reporting Items for Scoping Reviews (PRISMA-ScR)’ was adopted [17] (Annexure 1). The protocol was registered on Open Science Framework (OSF) and the link to the registration is as follows https://osf.io/cgd6e/.

The framework comprises 5 steps as detailed below:

**1. Identifying the research question**

What are the factors impacting healthcare service accessibility for older adults in the WHO - Southeast Asia?

This study adopted the PCC (Population, Concept, Context) format, according to the JBI manual for evidence synthesis 2020, for developing the research question [18] (Table 1).

**Table 1:** PCC framework for the research question

PCC Element	Description
Population (P)	Older adults (≥ 60 years, or as defined in individual studies)
Concept (C)	Factors influencing accessibility to healthcare services (e.g., barriers, facilitators, determinants, enablers, challenges, utilization patterns, perceptions)
Context (C)	WHO–Southeast Asia Region (SEAR) countries: Bangladesh, Bhutan, DPR Korea, India, Indonesia, Maldives, Myanmar, Nepal, Sri Lanka, Thailand, Timor-Leste

**2. Identifying relevant studies**

A comprehensive search was carried out for finding out for published literature in four databases: Scopus, PubMed, Web of Science, and CINHAL Ultimate using the keywords ‘older adults’, health care access’ and ‘WHO SEARO region’. A detailed search strategy was developed (Annexure 2) by the first author (AAK and AM) in consultation with subject experts and Boolean operators (AND/OR) were used to optimise search results. This study limited the date of publications from January 2014 to July 2025.

**3. Study selection**

**Fig. 1:**
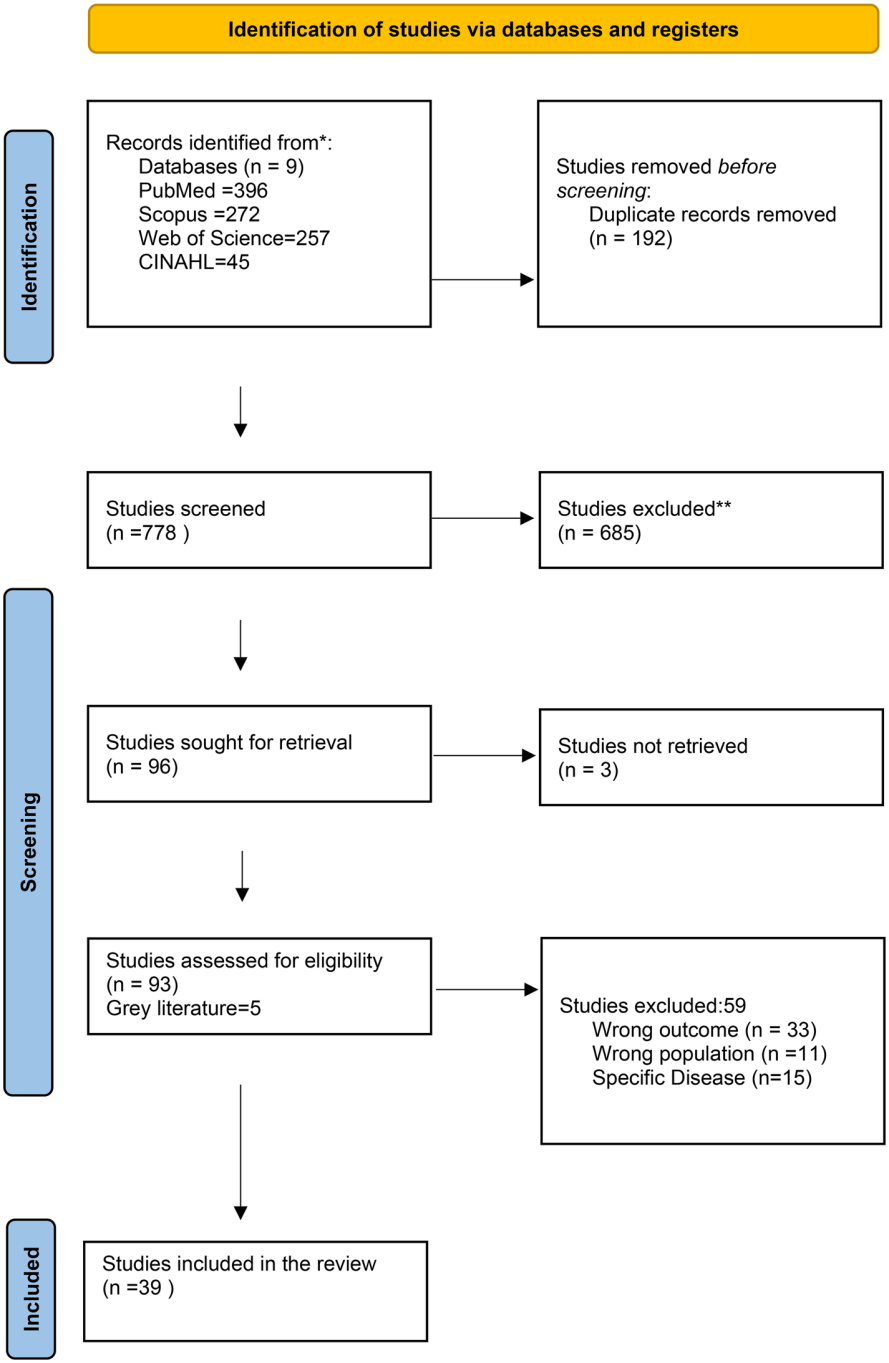
PRISMA 2020 flow diagram illustrating the systematic review process, including identification, screening, eligibility, and inclusion of studies for the review. *Consider, if feasible to do so, reporting the number of records identified from each database or register searched (rather than the total number across all databases/registers). **If automation tools were used, indicate how many records were excluded by a human and how many were excluded by automation tools

Study selection was performed by using Rayyan, a web-based platform [19]. Title and Abstract screening were performed independently by three reviewers, AAK, AM and RK. In cases of uncertainty, a fourth reviewer (PR or TAJJ) was consulted to reach consensus. The fourth author examined the rationale for exclusion or inclusion in cases of disagreement and determined which of the initial decisions best aligns with the agreed-upon protocol. Using the adjudication, the discretion of the fourth reviewer is considered final. Full texts of potentially eligible studies were then retrieved and assessed independently by two reviewers (AAK and AM). Studies meeting the eligibility criteria underwent data charting, and the overall selection process was documented using a PRISMA 2020 flow diagram [20] (Fig. 1). All studies fulfilling the pre-defined inclusion criteria were considered for the review, regardless of methodological quality (Table 2). The review included a range of study designs—quantitative, qualitative, and mixed-methods. To ensure comprehensive coverage, the reference lists of included studies were also examined to identify additional relevant publications.

**Table 2:** Inclusion and exclusion criteria

Inclusion criteria	Exclusion criteria
Studies focused on factors associated with ensuring healthcare service accessibility. Studies that explore healthcare service accessibility issues, including healthcare facilities, primary care, hospital care, long-term care, community care, home care, and related services for older adults.	Studies focus solely on specific diseases or conditions without considering healthcare service accessibility as a primary outcome.
Studies focusing on older adults (typically aged 60 and above).	Studies with insufficient information or lack of relevance to the research question.
Only original studies were included.	
Studies conducted in the WHO SEARO.	
Studies published in the English language.	

**4. Charting the data**

Data Extraction was performed by AAK, AM and RK adopting a predefined data charting form using Microsoft Excel.

**5. Collating summarizing**,** and reporting**

Extracted data were synthesized narratively, and the discussion was guided by the “**Levesque conceptual framework for healthcare access**. AAK and AM independently performed quality checks and reviews, adopting the PRISMA 2020 reporting guideline for scoping and systematic reviews [20].

## Results

The comprehensive literature search yielded 970 results, and 192 duplicates were removed. The title-abstract screening was done for 778 articles, resulting in the removal of 685 articles and ultimately, 93 articles were included for full-text screening. After full-text screening, 34 articles were included. Additionally, 5 relevant articles were identified from the references and included as grey literature, resulting in 39 articles for the study. Furthermore, detailed information for each included study is provided in the form of a study characteristics table (Annexure 3). The included studies underwent data charting for the final scoping review, detailed in a flowchart following the PRISMA 2020 diagram (Fig. 1).

Study populations predominantly comprised community-dwelling older adults, though some studies focused on institutionalized populations or specific subgroups (e.g., those with chronic conditions). Considerable heterogeneity was observed in sample sizes, outcome measures, and analytical frameworks. Despite this variation, a common thematic orientation emerged, with most studies addressing determinants of healthcare access from either a system-level perspective (infrastructure, financing, service delivery) or a non-system perspective (sociocultural, demographic, and household dynamics).

Careful examination of the findings indicated that they could be systematically organized into positive and negative factors to healthcare accessibility. These determinants were further classified into healthcare system–related and non-healthcare system–related domains, thereby providing a structured basis for synthesis. Importantly, this categorization enabled the consolidation of fragmented evidence into coherent thematic clusters, which in turn informed the development of a comprehensive conceptual framework presented in the subsequent section.

## Synthesis of empirical evidence: positive and negative outcomes

The synthesis of findings is classified as positive outcomes and negative outcomes across the factors related to the health system and the non-healthcare system, summarising enablers and barriers in health care access among older adults in WHO-SEARO countries.

## Positive outcomes: healthcare system-related

Across the 39 included studies, positive outcomes in healthcare system factors were evident in 23 studies [21–43]. Key themes included financial protections, infrastructural availability, and targeted programs, and reduced out-of-pocket expenditures (OOPE) for the older adults.

## Financial protection through insurance and subsidies

Insurance schemes emerged as a dominant enabler, mitigating affordability barriers and promoting equitable access in 16 studies [21–24, 30, 32–34, 36, 39–41, 44–47]. National programs like India’s Rashtriya Swasthya Bima Yojana (RSBY), Ayushman Bharat Pradhan Mantri Jan Arogya Yojana (AB-PMJAY), and publicly funded health insurance (PFHI) provided risk protection for the population, especially among the poorest quintile and immobile elderly [23, 38, 39, 41]. In Thailand, the Universal Coverage Scheme (UCS) and Civil Servant Medical Benefit Scheme facilitated free medications, reimbursements. It helped to achieve better utilization for chronic non-communicable diseases (NCDs), benefiting urban and rural elderly alike [21, 33, 42, 45]. Indonesia’s National Health Insurance covered 62.3% of the population, associating with increased outpatient service use among those with chronic conditions [46, 47]. Nepal’s social health insurance (SHI) and free premium provisions boosted facility visits by enhancing awareness and independence in daily activities [25, 34].

## Infrastructure and service availability

India’s primary health centres (PHCs), community health centres (CHCs), and sub-centres offered free or subsidized services, increasing hospitalization rates and utilization for acute, chronic, and inpatient needs among tribal and low-income elderly [22, 23, 31, 36, 40, 48, 49]. In Bangladesh, widespread primary care infrastructure reduced spatial gaps for older adults, serving as the main access point for low-cost services despite urban-rural disparities [50, 51]. Thailand’s nationwide primary health centers and district hospitals, coupled with equitable geographical distribution of secondary/tertiary facilities, ensured broad coverage and strong referral systems for NCD management [21, 42].

### National programs and community interventions

India’s National Programme for Health Care of the Elderly (NPHCE) and Health and Wellness Centres (HWCs) under Ayushman Bharat enhanced geriatric infrastructure, providing expanded service and preferential treatment for elderly and tribal groups, which improved timely access and health-seeking behaviors [26, 30–32, 49]. Community health workers, such as Accredited Social Health Activists (ASHAs) and Auxiliary Nurse Midwives (ANMs), bridged gaps through regular village visits and mobile services, fostering utilization among hard-to-reach populations [26, 31]. In Nepal and Bangladesh, government subsidies for severe conditions and free primary services further supported chronic care, reducing inequities for multimorbid older adults [25, 27].

These healthcare system enablers collectively help to reduce the unmet needs and financial burden among geriatric population.

## Positive outcomes: non-healthcare related

Non-healthcare system determinants substantially enable healthcare access among older adults across WHO–Southeast Asia (*n* = 26 [22, 24, 25, 29–43, 45–47], ]). These enablers, includes socioeconomic, familial, educational, and environmental determinants, facilitated healthcare access and utilization among older adults in SEARO countries, often intersecting with system-level supports.

## Socioeconomic status and economic independence

Higher socioeconomic status emerged as a key enabler, enhancing affordability and care-seeking beyond insurance. Elevated income and wealth quintiles correlated with increased utilization [23]. It also correlates with preference for private services [36, 48, 49]In Nepal, healthcare utilization was higher for older adults with high income [25]. Economic independence boosted insurance uptake and overall outpatient visits in Indonesia [46, 47]. Remittances from migrant children further supported functional ability and chronic morbidity management in India [52] while self-financing via spousal or familial aid reduced reliance on borrowing [29].

### Education and health literacy

Educational attainment fostered awareness and proactive behaviors, empowering older adults to seek timely care. Higher schooling years associated with treatment-seeking and improving health literacy and better system navigation [32, 36, 49, 53]. In Thailand, the health awareness and perceived necessity increased the healthcare utilization among urban population [33]. In India, Literacy enabled better multimorbidity management and private care access [40, 43].

### Family and social support

Sons and spouses are major source for both inpatient and outpatient care. And co-residence increased treatment-seeking by 25–40%, particularly in rural India and Nepal [29, 31, 34]. Married older adults preferred private care compared to widowed or divorced, with children accompanying visits raising [25, 43, 48]. Moral obligations for male adult support in Bangladesh and reduced isolation and difficulties for multimorbid elderly [38, 54]. Community ties, volunteer networks, and social protection programs further bridged gaps, enhancing awareness and enrollment in rural settings [30, 40, 42].

### Living arrangements and environmental enablers

Favorable living and locational factors supported mobility and proximity. Urban residence correlated with higher utilization and provider options, mitigating rural disparities [22, 32], ]. Not living alone and nuclear setups increased the chance of accessibility, especially in high-expectancy states [23, 38]. Transport options like rickshaws and proximity in some areas facilitated primary care, while community clinics offered localized preventive access despite limitations [33, 50].

### Negative outcomes related to healthcare system factors

Older adults in Southeast Asia encounter multiple healthcare-related barriers that constrain access and utilization, with significant consequences for health and well-being.

### Affordability barriers and financial hardships

Affordability challenges persisted despite insurance expansions, as highlighted in 34 studies [21–23, 25–44, 47, 48, 51, 52, 55–60], driving catastrophic expenditures. In India, OOPE averaged Rs. 7,573 annually for older adults, over twice the general population average and contributed to impoverishment rates 3% higher for those aged 60+ [60]. Insurance schemes like publicly funded health insurance (PFHI) covered only 18.9% of elderly overall, with critical gaps in outpatient and rehabilitative services, forcing reliance on high-cost private care (57.3% utilization) and borrowing/selling assets among the poorest people [23, 36–38]. Similar patterns emerged in Bangladesh and Nepal, where out-of-pocket medical expenses and transportation costs prevented routine NCD care, with 23% of multimorbid older adults facing medicine access difficulties [25, 54]. In Thailand, non-affordability of treatments, even under Universal Coverage Scheme (UCS), affected urban poor and elderly, amplifying pro-rich biases in visits and expenses [21, 42]. Indonesia reported lower coverage (62.3%) among rural and poorer elderly, correlating with reduced outpatient use [46].

### Accessibility and geographical disparities

Geographical and logistical barriers impeded access in several studies, particularly in rural settings, fostering unmet needs up to 58% in states like Meghalaya [38]. Rural-urban divides were stark, with non-accessibility in rural India limiting utilization and contributing to higher hospitalization in urban areas [22, 48]. In Bangladesh, geographically inaccessible health facilities in rural regions increased travel times and inequities [50, 51, 58, 59]. Nepal’s distant facilities, coupled with transportation and lodging costs, reduced utilization among chronically ill elderly, a pre-existing issue worsened by COVID-19 resource shifts [25]. Thailand faced urban demand overload and rural mobility gaps, with long waiting lists and bed shortages further alienating elderly [21, 33]. For immobile elderly (7.5% prevalence in India), long distances and poor infrastructure compounded exclusion from rehabilitative services [37, 39].

### Availability and infrastructure shortfalls

Limited availability of services and resources was a recurring theme in 22 studies, undermining accommodation for geriatric needs. Public facilities in India suffered from inadequate infrastructure, staff shortages, and low investment, leading to underutilization (30.2%) despite affordability advantages [32, 36, 49]. Specialized geriatric care was scarce, with no dedicated services in union clinics and shortages of doctors/nurses (one doctor per week in some Bangladeshi facilities), equipment, and medications delaying diagnoses [31, 51, 58, 59]. In Nepal and Thailand, inadequate drugs/equipment and human resource limitations hindered essential NCD management, with implementation gaps in free services awareness exacerbating non-use [34, 61]. COVID-19 amplified these, converting tertiary centers and straining routine care [25, 54].

### Quality and acceptability issues

Perceived poor quality and inequitable treatment impacted acceptability. In India, public sector concerns like long waits, uncleanliness, privacy lacks, and maltreatment deterred use, while private dominance imposed financial strain [43, 49, 51]. Bangladesh reported staff disinterest, verbal abuse, and maternal-care bias neglecting elderly women, fostering mistrust [51, 62]. Pro-rich inequities persisted in Thailand’s UCS, with higher socioeconomic status predicting more visits despite universality [42]. Overall, lower-quality care at primary levels and accountability gaps led to dissatisfaction in services among frail, multimorbid elderly [27, 30, 40].

These healthcare system barriers collectively perpetuate horizontal inequities, with rural and low-income older adults facing 40–50% higher unmet needs [28, 44].

### Negative outcomes related to non-healthcare system factors

Non-healthcare factors substantially constrain healthcare utilization among older adults in Southeast Asia (*n* = 28 [21–26, 28, 31–43, 48, 51, 53, 55, 58, 59, 61, 63]).

### Socioeconomic and poverty barriers

Economic vulnerability was a prevalent barrier to utilization, driving financial strain. Low income and poverty forced borrowing, asset sales, or reliance on inadequate pensions, with 70% economic dependence amplifying risks for multimorbid cases [23, 31, 37–39, 49]. In rural Nepal and Bangladesh, agricultural-based low earnings and lack of savings deterred visits (54.6% utilization rate), prioritizing family over personal needs [34, 54, 58]. Informal workers faced income loss from treatment absences, while urban poor encountered opportunity costs [39, 42, 50]. Poorer groups reported 40–50% higher unmet needs, with informal sector risks exacerbating NCD delays [43, 49].

### Educational and health literacy deficits

Low education and awareness gaps undermined proactive seeking, fostering misconceptions and delays. Illiteracy correlated with underuse of preventive services and higher unmet needs [32, 36, 38, 49]. In Bangladesh, lack of literacy led to inability to comprehend information, reliance on self-medication, and superstitions [56, 58, 59]. Rural residence reduced treatment, while low awareness of free services persisted as a “luxury” barrier in Nepal [25, 53]. Thailand linked with low education to fewer visits despite needs [42]. Similarly, 26.9% people with no formal education in rural Odisha, India suppressing formal care [40].

### Family and social support gaps

Deficient familial networks isolated vulnerable elderly, preventing support and emotional aid. Living alone and widowhood elevated unmet needs and borrowing, with migration leaving parents without support [23, 38, 48, 52]. Dysfunctional families reduced utilization in rural Nepal [34], while neglect/abuse from intergenerational shifts and resource limits prevailed in India [26, 61]. In Bangladesh, patriarchal dependencies and prioritization of youth/males marginalized women, fostering isolation [51, 54, 62]. Lack of companions and poor networks deterred care, especially for disabled [21, 25, 40, 42].

### Cultural, gender, and environmental factors

Lack of perceived need (61.1%) due to age, fear (6.6% for surgery), or “no one to accompany” (20.3%) dominated person-related issues, alongside traditional medicine preferences [25, 31, 55, 59]. Gender disparities hit women harder, with widowhood, low status, and multi-dimensional barriers raising disability risks [42, 43, 48, 49]. Rural residence showed less utilization than urban and poor transport lead to worsening for immobile [22, 32, 37, 50, 53]. Social isolation and cultural dissonance further eroded trust in formal systems [26, 31, 40]. These non-healthcare barriers intensify disparities, with rural, female, and low socio economic status.

## Discussion

**Fig. 2:**
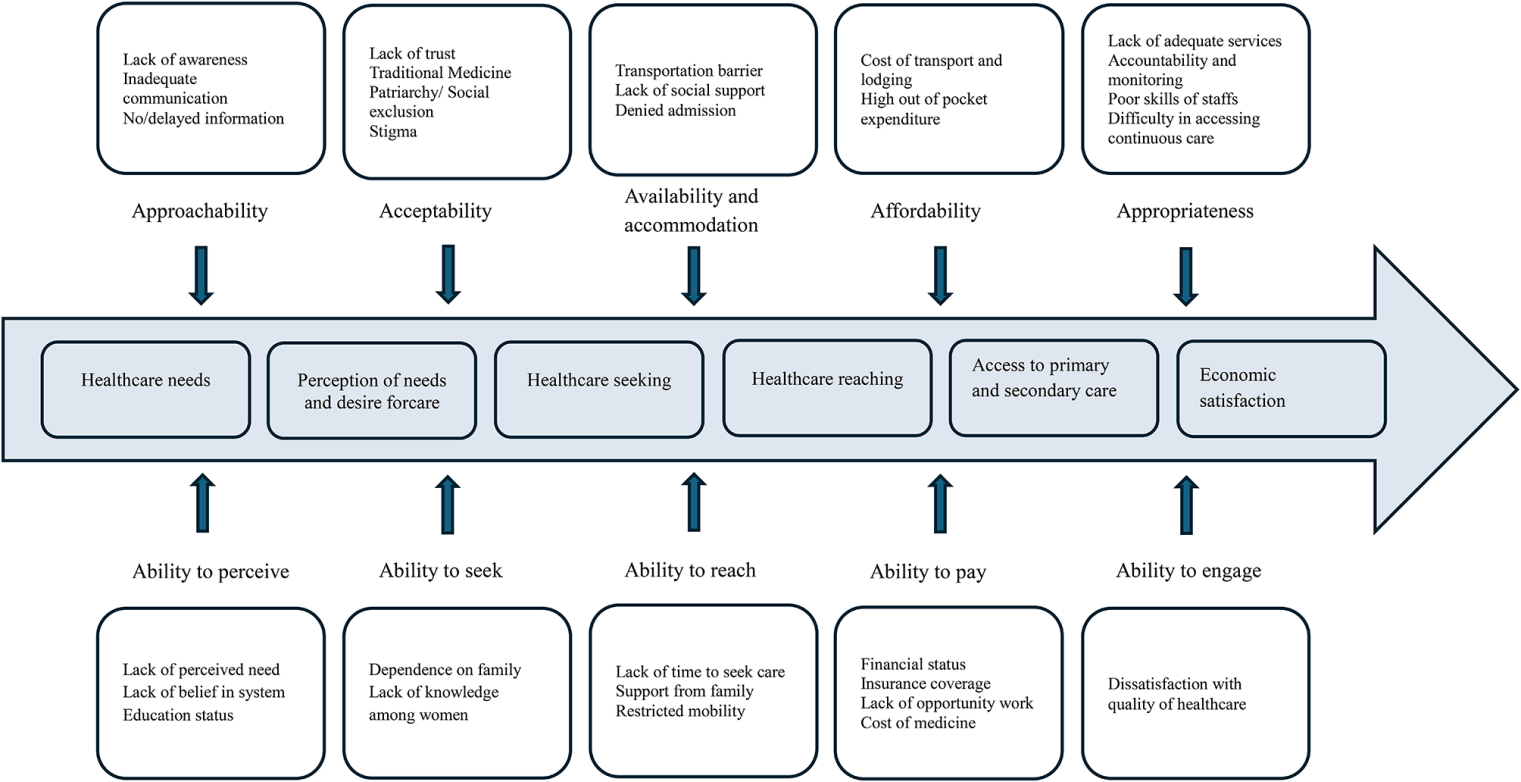
Adapted Levesque conceptual framework for healthcare access among older adults in WHO-SEARO

This scoping review highlights that healthcare access for older adults in Southeast Asia is constrained by both system-related and individual-level determinants, which can be coherently interpreted through adopting the “Levesque conceptual framework for healthcare access” as summarised in Fig. 2 [15]. Older adults across Southeast Asia encounter intersecting barriers to healthcare access spanning financial, geographic, social, and systemic domains. Financial constraints remain the most pervasive determinant, as out-of-pocket payments for consultations, medications, and transportation often delay or diminish care access [51]. Although health insurance remains pivotal in managing out-of-pocket expenditure, contradictory evidence has been reported from LMICs on poor outpatient and rehabilitative coverage, underscoring the need for stronger social health insurance and benefit packages tailored to older populations [36, 42, 47].

In line with the findings from comparable settings [64, 65], our analysis on barriers to health care access reported that long travel distances, costly or unavailable transport, and shortages of trained providers rurality and limited mobility often intersect with age-related frailty to reduce service use [61]. These disparities could be addressed through effective Policy efforts, such as decentralisation of geriatric care, community-based outreach, and mobile health initiatives, which mitigate these spatial inequities [25, 26, 28, 51, 66, 67]. While the present study highlighted how the process of access to health services accessibility is influenced, how the healthcare agencies have adopted immediate measures to respond to the ongoing disparity, and how the factors have affected the dissemination of scientific information. The findings on various factors that intervene with the health care utilisation pattern among older adults are discussed and categorised into the following themes: approachability, acceptability, availability and accommodation, affordability, appropriateness, ability to perceive, ability to seek, ability to reach, ability to pay and ability to engage by the classification by Levesque’s conceptual framework for healthcare access to healthcare.

### Acceptability

Older adults encounter issues related to acceptability in healthcare settings. They perceived a lack of interest and engagement from healthcare staff in providing care. This sentiment is further echoed by patients who express dissatisfaction stemming from the absence of robust systems for accountability and monitoring. Additionally, older adults report a prevailing negative attitude from healthcare professionals toward This is contrary to the findings of a systematic review conducted from 2000 to 2011, which showed a positive attitude toward older adults by healthcare professionals [68]. This disparity in attitude could be due to the lack of a proper geriatric care system in Southeast Asian countries. Existing evidence on accessibility barriers also reports disrespectful provider attitudes, limited communication, and a lack of accountability, which causes eroded trust in the health system services [26, 51]. Comparable studies in other regions also document ageism and inadequate geriatric training as barriers to utilization [66, 67]. Addressing these deficits requires integrating geriatric care into professional curricula, strengthening provider–patient communication, and ensuring accountability mechanisms that foster patient-centred and dignified care [25, 28].

### Approachability

In a health system, services are available but less well-known among the population due to various intervening factors. In India, the provision of health services has been enhanced by mobile health units, with 104 mobile health services specifically catering to the healthcare needs of older adults [26]. Despite these efforts, challenges persist. The predominant issues include both the lack of awareness regarding available services and purposeful ignorance towards health concerns [21, 25, 33, 69]. Marginalization within patient-staff relationships further worsens the problem [51, 69]. Findings from India consolidated that health-seeking behaviour was low (28%), exacerbated by cultural barriers and limited access, where women showed 20% lower awareness due to gender disparities. These disparities could be further exacerbated by non-health system-related factors such as education, family support and rural-urban divide. Comparable findings from Bangladesh also indicated that increasing awareness of the health system often enhances service utilisation among olde adults [70, 71].

### Availability and accommodation

Public care provisions, including medical camps and proximity to healthcare facilities, play a crucial role in ensuring accessible healthcare services. People with chronic diseases and belonging to lower socioeconomic groups experience higher levels of barriers related to transportation [72, 73]. In India, challenges in accessing diagnostic services remain preponderant for older adults. Factors such as frailty, limited mobility, and dependence on family members for transportation contribute to these access barriers [26]. Furthermore, the unequal distribution of health services across regions aggravates the healthcare access disparities. In Bangladesh, the healthcare infrastructure faces several challenges, including an inadequate number of public healthcare facilities and insufficient facilities within health centres [51].

### Affordability

Studies have highlighted that older adults experience added financial burdens while accessing health services [27]. In Nepal, the government’s initiative to provide free primary health services and treatment subsidies for severe health conditions among older adults has been implemented [57]. In India, several public healthcare schemes, such as Aarogyasri and Rashtriya Swasthya Bima Yojana, offer free or low-cost healthcare services to the population. While there have been positive developments in India, with increased coverage of the insured population attributed to various national initiatives like the Rashtriya Swasthya Bima Yojana and subnational policies like the Arogyashree Scheme (28), income-related inequities and inequalities in healthcare utilization remain prominent concerns for the well-being of older adults [28, 74]. Despite these initiatives, OOPE are notably higher among individuals with disabilities and those with lower income levels [27]. The persistent requirement for substantial OOPE may compel disabled, economically disadvantaged, and older individuals to either forgo or underutilize healthcare services [27]. The intersection of economic independence and other socioeconomic factors intensifies these disparities. Financially empowered and better-off older adults are likely to experience better health outcomes and require less inpatient care compared to their economically disadvantaged counterparts [75].

### Appropriateness

Though there are initiatives to complement health care for older adults, the holistic idea of geriatric care is yet to flourish. In Nepal, dissatisfaction with the quality of healthcare services has been identified as a significant deterrent to utilizing health services, even when they are provided free of charge [25]. Additionally, in India, the lack of specialized geriatric services further compounds the challenges faced by the older population in accessing appropriate and tailored healthcare services [56].

### Ability to perceive

Higher level of education is a crucial determinant in enhancing healthcare utilization among older adults in India [22, 76]. Lack of perceived need to seek healthcare interventions [55]. Insufficient information regarding healthcare management and treatment seeking is found to have a crucial impact on shaping the abilities to perceive healthcare beliefs and actions [21, 77]. Access to health-related information is positively correlated with the level of education among older adults [55].

### Ability to seek

The ability to seek health care is an intersection of various socio-economic factors that determine the capacity of older adults to obtain care when needed. The ability to seek care is highly influenced by gendered vulnerabilities, indicating higher deprivations among women [75]. However, Naz et al., [78] state that gender is not a significant indicator of healthcare utilization. Older women were found to have higher treatment seeking for lifestyle diseases, including diabetes and hypertension, when compared to their male counterparts indicating contractions in gender-based disparities of care, which is further confirmed by Naz et al., [78], stating that gender is an insignificant variable in terms of deciding the health care utilization patterns and choices. The gender-based deprivations are still a matter of debate, and the disparity could be due to the varying socio-cultural determinants and support structures, which vary from country to country. Economic dependency in old age, chronic disability, vulnerabilities due to socioeconomic categorization, and rural-urban dividend interfere with the ability of older adults to seek health care utilization in old age [22, 23, 27].

### Ability to reach

Older adults are attributed to declining mobility, implying the need for prioritizing of means to reach service providers physically. Living arrangements and family support are the major determinants that enhance the ability to access health care among older adults [22, 48]. Living alone is associated with added deprivation in care, indicating the need for someone to accompany them to the hospitals to ensure timely care and service access among older adults [79]. The availability of alternative and extended support systems and social networking could have a compensatory role in addressing the care deficit among older adults who are in solitary living.

### Ability to pay

The wealth status of the family and employment prospects of the older adults are decisive in assessing the ability to pay. However high cost of treatment, lack of insurance coverage, out-of-pocket expenditures, and cost of transportation interfere with the ability to pay for and afford healthcare facilities among older adults. As confirmed by the previous literature, having health insurance increases the health care utilization among older adults, irrespective of their health needs, whereas having no insurance reduces the help-seeking patterns [63]. Previous literature confirms that the vulnerable population relies more on public healthcare facilities, implying the need for targeted interventions for older adults belonging to lower economic strata and socially fragile communities [80]. Increasing health insurance coverage can enhance the level of health care utilization among older adults, irrespective of their socioeconomic disparities.

### Ability to engage

The fit between needs and quality care is conceptualized as the ability to engage. Satisfaction with available interventions and quality of care measures the health engagement in terms of appropriateness. In line with the findings, Ferreira et al., [81] argues that the prolonged waiting time, financial stress of treatment, lack of trust in the care offered, behavior of staff, and distance to the place which offers treatment are decisive in the ability to bridge the care gap among older adults. In congruent with inverse care law [27]. Older adults who need advanced care take in the lowest care provisions owing to various factors that impede healthcare utilisation.

### Strengths and limitations

One notable strength is the adoption of the Levesque conceptual framework for healthcare access to healthcare, which distinguishes this study and facilitates policymakers in implementing targeted interventions to enhance healthcare accessibility. Nevertheless, the study is subject to limitations as it is limited to four databases that exclusively incorporate English literature, thereby potentially overlooking relevant literature available in other regional languages. Further, the search was limited to four databases and WHOSEARO countries which might have excluded important local evidence, implying the need for more comprehensive research focusing on other middle-income and low-income countries. Additionally, scoping review methodology falls short in measuring causality or intervention effectiveness, suggesting systematic and meta-analysis to gather effective interventions to enhance healthcare accessibility among older adults.

## Conclusion

WHO-SEARO countries have made tangible progress in improving healthcare service delivery among older adults through various targeted interventions. The critical analysis of facilitators and barriers to healthcare accessibility reveals the interplay between health perceptions and health-seeking behavior. Despite the advancements in extending health care utilization across different age groups, inadequate health information, increased economic dependency on children, reduced accessibility due to distance and cost of travel, and high out-of-pocket expenditure, solitary living patterns are found to be the major barriers in health care utilisation among older adults. Addressing these challenges, particularly within the WHO South-East Asia Region, requires the immediate adoption of Integrated Person-Centred Care (ICOPE) at the community level, expanding insurance coverage, improving geriatric training, and strengthening rural outreach programs. Future research should focus on integrating long-term digital health interventions in mitigating rural-urban care disparities by informing evidence-based policy recommendations for equitable care for this rapidly growing population of older adults.

## Supplementary Information

Below is the link to the electronic supplementary material.

Supplementary Material 1: Annexure 1: PRISMA-ScR Check list.

Supplementary Material 2: Annexure 2: Search strategy.

Supplementary Material 3: Annexure 3: Characteristic table.

## Data Availability

No datasets were generated or analysed during the current study.

## Abbreviations

ABPMJAY: Ayushman Bharat and Pradhan Mantri Jan Arogya Yojana
CGHS: Central Government Health Scheme
CHC: Community Health Centre
ESI: Employees State Insurance Scheme
NGO: Non-Governmental Organisation
OPPE: Out of Pocket Expenditure
PCC: Population Context Framework
PFHI: Publicly funded health insurance
PHC: Primary Health Centre
PRISMA: Preferred Reporting Items for Systematic reviews and Meta-Analyses
SDG: Sustainable Development Goals
WHO SEARO: World Health Organisation South East Asia Regional Office
WHO: World Health Organisation
