# Supplementary material for: Factors influencing healthcare access among older adults in Southeast Asia: a scoping review guided by the levesque framework
Source: BMC Health Serv Res. 2025 Dec 7;26:55. doi: 10.1186/s12913-025-13838-8 (PMC12797660; doi:10.1186/s12913-025-13838-8)
Supplement: Supplementary file 2 — Supplementary Material 2: Annexure 2: Search strategy. [file 12913_2025_13838_MOESM2_ESM.docx]

| **Concepts for formulating search strategies** | |
| --- | --- |
| **Concept** | **Search terms** |
| Older adults | “Older adults” OR Elderly OR Aged OR “Senior citizens” OR “Geriatric population” OR “Aging population” OR “Elder population” OR “Older population” OR “Older individuals” OR “Aging adults” OR “Elderly people” OR “Senior adults” OR “Geriatric adults” OR “Aging seniors” OR “Advanced age group” OR “Older men and women” OR “Mature adults” OR “Senior population” OR “Older age group” OR “Older persons” |
| Access to Healthcare | "Healthcare accessibility" OR "Health services accessibility" OR "Healthcare availability" OR "Health system access" OR "Medical care access" OR "Healthcare utilization" OR "Health disparities" OR "Health equity" OR "Healthcare disparities" OR "Barriers to healthcare" OR "Health access barriers" OR "Healthcare utilization patterns" OR "Health insurance coverage" OR "Healthcare delivery" OR "Equity in healthcare" OR "Health resource allocation" OR "Healthcare provision" OR "Health service availability" |
| Southeast Asia | "WHO SEAR" OR Bangladesh OR Bhutan OR India OR Maldives OR Nepal OR Pakistan OR Sri Lanka OR “Timor Leste” OR Myanmar OR Thailand OR “North Korea” OR Indonesia |

Annexure 2: Search strategy

| PubMed: 31/07/25 | | |
| --- | --- | --- |
| Concept | Search Strategy | Hits |
| Older adults | "Older adults"[Title/Abstract] OR "Elderly"[Title/Abstract] OR "Aged"[Title/Abstract] OR "Senior citizens"[Title/Abstract] OR "Geriatric population"[Title/Abstract] OR "Aging population"[Title/Abstract] OR "Elder population"[Title/Abstract] OR "Older population"[Title/Abstract] OR "Older individuals"[Title/Abstract] OR "Aging adults"[Title/Abstract] OR "Elderly people"[Title/Abstract] OR "Senior adults"[Title/Abstract] OR "Geriatric adults"[Title/Abstract] OR "Aging seniors"[Title/Abstract] OR "Advanced age group"[Title/Abstract] OR "Older men and women"[Title/Abstract] OR "Mature adults"[Title/Abstract] OR "Senior population"[Title/Abstract] OR "Older age group"[Title/Abstract] OR "Older persons"[Title/Abstract] OR "Health Services for the Aged"[MeSH Terms] | [688,828](https://pubmed.ncbi.nlm.nih.gov/?term=%22Older+adults%22%5BTitle%2FAbstract%5D+OR+%22Elderly%22%5BTitle%2FAbstract%5D+OR+%22Aged%22%5BTitle%2FAbstract%5D+OR+%22Senior+citizens%22%5BTitle%2FAbstract%5D+OR+%22Geriatric+population%22%5BTitle%2FAbstract%5D+OR+%22Aging+population%22%5BTitle%2FAbstract%5D+OR+%22Elder+population%22%5BTitle%2FAbstract%5D+OR+%22Older+population%22%5BTitle%2FAbstract%5D+OR+%22Older+individuals%22%5BTitle%2FAbstract%5D+OR+%22Aging+adults%22%5BTitle%2FAbstract%5D+OR+%22Elderly+people%22%5BTitle%2FAbstract%5D+OR+%22Senior+adults%22%5BTitle%2FAbstract%5D+OR+%22Geriatric+adults%22%5BTitle%2FAbstract%5D+OR+%22Aging+seniors%22%5BTitle%2FAbstract%5D+OR+%22Advanced+age+group%22%5BTitle%2FAbstract%5D+OR+%22Older+men+and+women%22%5BTitle%2FAbstract%5D+OR+%22Mature+adults%22%5BTitle%2FAbstract%5D+OR+%22Senior+population%22%5BTitle%2FAbstract%5D+OR+%22Older+age+group%22%5BTitle%2FAbstract%5D+OR+%22Older+persons%22%5BTitle%2FAbstract%5D+OR+%22Health+Services+for+the+Aged%22%5BMeSH+Terms%5D&sort=&filter=dates.2014%2F7%2F1-2025%2F7%2F31) |
| Access to Healthcare | "Healthcare accessibility"[Title/Abstract] OR "health services accessibility"[Title/Abstract] OR "Healthcare availability"[Title/Abstract] OR "Health system access"[Title/Abstract] OR "Medical care access"[Title/Abstract] OR "Healthcare utilization"[Title/Abstract] OR "Health disparities"[Title/Abstract] OR "Health equity"[Title/Abstract] OR "Healthcare disparities"[Title/Abstract] OR "Barriers to healthcare"[Title/Abstract] OR "Health access barriers"[Title/Abstract] OR "Healthcare utilization patterns"[Title/Abstract] OR "Health insurance coverage"[Title/Abstract] OR "Healthcare delivery"[Title/Abstract] OR "Equity in healthcare"[Title/Abstract] OR "Health resource allocation"[Title/Abstract] OR "Healthcare provision"[Title/Abstract] OR "Health service availability"[Title/Abstract] OR "health services accessibility"[MeSH Terms] | [102,742](https://pubmed.ncbi.nlm.nih.gov/?term=%22Healthcare+accessibility%22%5BTitle%2FAbstract%5D+OR+%22health+services+accessibility%22%5BTitle%2FAbstract%5D+OR+%22Healthcare+availability%22%5BTitle%2FAbstract%5D+OR+%22Health+system+access%22%5BTitle%2FAbstract%5D+OR+%22Medical+care+access%22%5BTitle%2FAbstract%5D+OR+%22Healthcare+utilization%22%5BTitle%2FAbstract%5D+OR+%22Health+disparities%22%5BTitle%2FAbstract%5D+OR+%22Health+equity%22%5BTitle%2FAbstract%5D+OR+%22Healthcare+disparities%22%5BTitle%2FAbstract%5D+OR+%22Barriers+to+healthcare%22%5BTitle%2FAbstract%5D+OR+%22Health+access+barriers%22%5BTitle%2FAbstract%5D+OR+%22Healthcare+utilization+patterns%22%5BTitle%2FAbstract%5D+OR+%22Health+insurance+coverage%22%5BTitle%2FAbstract%5D+OR+%22Healthcare+delivery%22%5BTitle%2FAbstract%5D+OR+%22Equity+in+healthcare%22%5BTitle%2FAbstract%5D+OR+%22Health+resource+allocation%22%5BTitle%2FAbstract%5D+OR+%22Healthcare+provision%22%5BTitle%2FAbstract%5D+OR+%22Health+service+availability%22%5BTitle%2FAbstract%5D+OR+%22health+services+accessibility%22%5BMeSH+Terms%5D&sort=&filter=dates.2014%2F7%2F1-2025%2F7%2F31) |
| WHO SEAR | "South Asia"[Title/Abstract] OR "Bangladesh"[Title/Abstract] OR "Bhutan"[Title/Abstract] OR "India"[Title/Abstract] OR "Maldives"[Title/Abstract] OR "Nepal"[Title/Abstract] OR "Sri Lanka"[Title/Abstract] OR "Indonesia"[Title/Abstract] OR "Myanmar"[Title/Abstract] OR "Timor Leste"[Title/Abstract] OR "Thailand"[Title/Abstract] OR "North Korea"[Title/Abstract] | [165,891](https://pubmed.ncbi.nlm.nih.gov/?term=%22South+Asia%22%5BTitle%2FAbstract%5D+OR+%22Bangladesh%22%5BTitle%2FAbstract%5D+OR+%22Bhutan%22%5BTitle%2FAbstract%5D+OR+%22India%22%5BTitle%2FAbstract%5D+OR+%22Maldives%22%5BTitle%2FAbstract%5D+OR+%22Nepal%22%5BTitle%2FAbstract%5D+OR+%22Sri+Lanka%22%5BTitle%2FAbstract%5D+OR+%22Indonesia%22%5BTitle%2FAbstract%5D+OR+%22Myanmar%22%5BTitle%2FAbstract%5D+OR+%22Timor+Leste%22%5BTitle%2FAbstract%5D+OR+%22Thailand%22%5BTitle%2FAbstract%5D+OR+%22North+Korea%22%5BTitle%2FAbstract%5D&sort=&filter=dates.2014%2F7%2F1-2025%2F7%2F31) |
| #1 AND #2 AND #3 | ("Older adults"[Title/Abstract] OR "Elderly"[Title/Abstract] OR "Aged"[Title/Abstract] OR "Senior citizens"[Title/Abstract] OR "Geriatric population"[Title/Abstract] OR "Aging population"[Title/Abstract] OR "Elder population"[Title/Abstract] OR "Older population"[Title/Abstract] OR "Older individuals"[Title/Abstract] OR "Aging adults"[Title/Abstract] OR "Elderly people"[Title/Abstract] OR "Senior adults"[Title/Abstract] OR "Geriatric adults"[Title/Abstract] OR "Aging seniors"[Title/Abstract] OR "Advanced age group"[Title/Abstract] OR "Older men and women"[Title/Abstract] OR "Mature adults"[Title/Abstract] OR "Senior population"[Title/Abstract] OR "Older age group"[Title/Abstract] OR "Older persons"[Title/Abstract] OR "Health Services for the Aged"[MeSH Terms]) AND ("Healthcare accessibility"[Title/Abstract] OR "health services accessibility"[Title/Abstract] OR "Healthcare availability"[Title/Abstract] OR "Health system access"[Title/Abstract] OR "Medical care access"[Title/Abstract] OR "Healthcare utilization"[Title/Abstract] OR "Health disparities"[Title/Abstract] OR "Health equity"[Title/Abstract] OR "Healthcare disparities"[Title/Abstract] OR "Barriers to healthcare"[Title/Abstract] OR "Health access barriers"[Title/Abstract] OR "Healthcare utilization patterns"[Title/Abstract] OR "Health insurance coverage"[Title/Abstract] OR "Healthcare delivery"[Title/Abstract] OR "Equity in healthcare"[Title/Abstract] OR "Health resource allocation"[Title/Abstract] OR "Healthcare provision"[Title/Abstract] OR "Health service availability"[Title/Abstract] OR "health services accessibility"[MeSH Terms]) AND ("South Asia"[Title/Abstract] OR "Bangladesh"[Title/Abstract] OR "Bhutan"[Title/Abstract] OR "India"[Title/Abstract] OR "Maldives"[Title/Abstract] OR "Nepal"[Title/Abstract] OR "Sri Lanka"[Title/Abstract] OR "Indonesia"[Title/Abstract] OR "Myanmar"[Title/Abstract] OR "Timor Leste"[Title/Abstract] OR "Thailand"[Title/Abstract] OR "North Korea"[Title/Abstract] OR "WHO SEAR"[Title/Abstract]) | 396 |
| Scopus date: 31/07/25 | | |
|  | TITLE-ABS ( "Older adults"  OR  elderly  OR  aged  OR  "Senior citizens"  OR  "Geriatric population"  OR  "Aging population"  OR  "Elder population"  OR  "Older population"  OR  "Older individuals"  OR  "Aging adults"  OR  "Elderly people"  OR  "Senior adults"  OR  "Geriatric adults"  OR  "Aging seniors"  OR  "Advanced age group"  OR  "Older men and women"  OR  "Mature adults"  OR  "Senior population"  OR  "Older age group"  OR  "Older persons" ) | 967,522 |
|  | TITLE-ABS ( "Healthcare accessibility"  OR  "Health services accessibility"  OR  "Healthcare availability"  OR  "Health system access"  OR  "Medical care access"  OR  "Healthcare utilization"  OR  "Health disparities"  OR  "Health equity"  OR  "Healthcare disparities"  OR  "Barriers to healthcare"  OR  "Health access barriers"  OR  "Healthcare utilization patterns"  OR  "Health insurance coverage"  OR  "Healthcare delivery"  OR  "Equity in healthcare"  OR  "Health resource allocation"  OR  "Healthcare provision"  OR  "Health service availability" ) | 57,490 |
|  | TITLE-ABS ( "WHO SEAR"  OR  bangladesh  OR  bhutan  OR  india  OR  maldives  OR  nepal  OR  pakistan  OR  "Sri Lanka"  OR  "Timor Leste"  OR  Myanmar OR Indonesia  OR  thailand  OR  "North Korea"  ) | 736,359 |
|  | ( TITLE-ABS ( "Older adults"  OR  elderly  OR  aged  OR  "Senior citizens"  OR  "Geriatric population"  OR  "Aging population"  OR  "Elder population"  OR  "Older population"  OR  "Older individuals"  OR  "Aging adults"  OR  "Elderly people"  OR  "Senior adults"  OR  "Geriatric adults"  OR  "Aging seniors"  OR  "Advanced age group"  OR  "Older men and women"  OR  "Mature adults"  OR  "Senior population"  OR  "Older age group"  OR  "Older persons" ) )  AND  ( TITLE-ABS ( "Healthcare accessibility"  OR  "Health services accessibility"  OR  "Healthcare availability"  OR  "Health system access"  OR  "Medical care access"  OR  "Healthcare utilization"  OR  "Health disparities"  OR  "Health equity"  OR  "Healthcare disparities"  OR  "Barriers to healthcare"  OR  "Health access barriers"  OR  "Healthcare utilization patterns"  OR  "Health insurance coverage"  OR  "Healthcare delivery"  OR  "Equity in healthcare"  OR  "Health resource allocation"  OR  "Healthcare provision"  OR  "Health service availability" ) )  AND  ( TITLE-ABS ( "WHO SEAR"  OR  bangladesh  OR  bhutan  OR  india  OR  maldives  OR  nepal  OR  pakistan  OR  "Sri Lanka"  OR  "Timor Leste"  OR  Myanmar OR Indonesia  OR  thailand  OR  "North Korea" ) ) | 272 |

| CINAHL date: 31/07/25 | | |
| --- | --- | --- |
| Older adults #S1 | TI ( “Older adults” OR Elderly OR Aged OR “Senior citizens” OR “Geriatric population” OR “Aging population” OR “Elder population” OR “Older population” OR “Older individuals” OR “Aging adults” OR “Elderly people” OR “Senior adults” OR “Geriatric adults” OR “Aging seniors” OR “Advanced age group” OR “Older men and women” OR “Mature adults” OR “Senior population” OR “Older age group” OR “Older persons” ) OR AB ( “Older adults” OR Elderly OR Aged OR “Senior citizens” OR “Geriatric population” OR “Aging population” OR “Elder population” OR “Older population” OR “Older individuals” OR “Aging adults” OR “Elderly people” OR “Senior adults” OR “Geriatric adults” OR “Aging seniors” OR “Advanced age group” OR “Older men and women” OR “Mature adults” OR “Senior population” OR “Older age group” OR “Older persons” ) | 392044 |
| Access to Healthcare #S2 | TI ( "Healthcare accessibility" OR "Health services accessibility" OR "Healthcare availability" OR "Health system access" OR "Medical care access" OR "Healthcare utilization" OR "Health disparities" OR "Health equity" OR "Healthcare disparities" OR "Barriers to healthcare" OR "Health access barriers" OR "Healthcare utilization patterns" OR "Health insurance coverage" OR "Healthcare delivery" OR "Equity in healthcare" OR "Health resource allocation" OR "Healthcare provision" OR "Health service availability" ) OR AB ( "Healthcare accessibility" OR "Health services accessibility" OR "Healthcare availability" OR "Health system access" OR "Medical care access" OR "Healthcare utilization" OR "Health disparities" OR "Health equity" OR "Healthcare disparities" OR "Barriers to healthcare" OR "Health access barriers" OR "Healthcare utilization patterns" OR "Health insurance coverage" OR "Healthcare delivery" OR "Equity in healthcare" OR "Health resource allocation" OR "Healthcare provision" OR "Health service availability" ) | 25045 |
| WHO SEAR #S3 | TI ( "WHO SEAR" OR Bangladesh OR Bhutan OR India OR Maldives OR Nepal OR Pakistan" OR "Sri Lanka" OR “Timor Leste” OR Myanmar OR Thiland OR “North Korea” ) OR AB ( "WHO SEAR" OR Bangladesh OR Bhutan OR India OR Maldives OR Nepal OR Pakistan" OR "Sri Lanka" OR “Timor Leste” OR Myanmar OR Thailand OR “North Korea” ) | 42864 |
| Combined S1 AND S2 And S3 | (TI "WHO SEAR" OR Bangladesh OR Bhutan OR India OR Maldives OR Nepal OR Pakistan" OR "Sri Lanka" OR “Timor Leste” OR Myanmar OR Thailand OR “North Korea” OR AB "WHO SEAR" OR Bangladesh OR Bhutan OR India OR Maldives OR Nepal OR Pakistan" OR "Sri Lanka" OR “Timor Leste” OR Myanmar OR Thailand OR “North Korea”) AND (S1 AND S2 AND S3) | 45 |
| WOS date: 31/07/25 | | |
| #1 Older adults | **(TI=(“Older adults” OR Elderly OR Aged OR “Senior citizens” OR “Geriatric population” OR “Aging population” OR “Elder population” OR “Older population” OR “Older individuals” OR “Aging adults” OR “Elderly people” OR “Senior adults” OR “Geriatric adults” OR “Aging seniors” OR “Advanced age group” OR “Older men and women” OR “Mature adults” OR “Senior population” OR “Older age group” OR “Older persons”)) OR AB=(“Older adults” OR Elderly OR Aged OR “Senior citizens” OR “Geriatric population” OR “Aging population” OR “Elder population” OR “Older population” OR “Older individuals” OR “Aging adults” OR “Elderly people” OR “Senior adults” OR “Geriatric adults” OR “Aging seniors” OR “Advanced age group” OR “Older men and women” OR “Mature adults” OR “Senior population” OR “Older age group” OR “Older persons”)** | 2,760,891 |
| # Access to Healthcare | **(TI=("Healthcare accessibility" OR "Health services accessibility" OR "Healthcare availability" OR "Health system access" OR "Medical care access" OR "Healthcare utilization" OR "Health disparities" OR "Health equity" OR "Healthcare disparities" OR "Barriers to healthcare" OR "Health access barriers" OR "Healthcare utilization patterns" OR "Health insurance coverage" OR "Healthcare delivery" OR "Equity in healthcare" OR "Health resource allocation" OR "Healthcare provision" OR "Health service availability")) OR AB=("Healthcare accessibility" OR "Health services accessibility" OR "Healthcare availability" OR "Health system access" OR "Medical care access" OR "Healthcare utilization" OR "Health disparities" OR "Health equity" OR "Healthcare disparities" OR "Barriers to healthcare" OR "Health access barriers" OR "Healthcare utilization patterns" OR "Health insurance coverage" OR "Healthcare delivery" OR "Equity in healthcare" OR "Health resource allocation" OR "Healthcare provision" OR "Health service availability")** | 48,136 |
| Countries | TI ( "WHO SEAR" OR Bangladesh OR Bhutan OR India OR Maldives OR Nepal OR Pakistan" OR "Sri Lanka" OR “Timor Leste” OR Myanmar OR Thiland OR “North Korea” ) OR AB ( "WHO SEAR" OR Bangladesh OR Bhutan OR India OR Maldives OR Nepal OR Pakistan" OR "Sri Lanka" OR “Timor Leste” OR Indonesia OR Myanmar OR Thailand OR “North Korea” ) | 226,714 |
| #1 AND #2 + Filter WHO SEAR | **#1 AND #2** and **BANGLADESH** or **BHUTAN** or **INDIA** or **NEPAL** or **PAKISTAN** or **SRI LANKA** or **MYANMAR** or **THAILAND** (Countries/Regions) | 257 |
