# Supplementary material for: Factors influencing healthcare access among older adults in Southeast Asia: a scoping review guided by the levesque framework
Source: BMC Health Serv Res. 2025 Dec 7;26:55. doi: 10.1186/s12913-025-13838-8 (PMC12797660; doi:10.1186/s12913-025-13838-8)
Supplement: Supplementary file 3 — Supplementary Material 3: Annexure 3: Characteristic table. [file 12913_2025_13838_MOESM3_ESM.docx]

| **No** | **Study Tittle** | **Authors & year** | **study setting** | **study design** | **Aims and objective** | **Findings of the study** | **Positive factors of the healthcare system in ensuring healthcare service accessibility** | **Negative factors of the healthcare system in ensuring healthcare service accessibility** | **Positive factors of the non-healthcare system in ensuring healthcare service accessibility** | **Negative factors of the non-healthcare system in ensuring healthcare service accessibility** | **Other findings** |
| --- | --- | --- | --- | --- | --- | --- | --- | --- | --- | --- | --- |
| 1 | A population-based cross-sectional study of barriers to uptake of eye care services in South India: The Rapid Assessment of Visual Impairment (RAVI) project | Srinivas Marmamula, Rohit C Khanna Konegari Shekhar, Gullapal N Rao, 2014 | India | Cross-Sectional Design | To assess the barriers to uptake of eye care services among those with avoidable impairment in the population aged ≥40 years in the South Indian state of Andhra Pradesh. | Prevalence of a person related barriers = 71.1%, (n=617) whereas reported service-related barriers is 28.9% (n=251). | NA | Lack of affordability was the major barrier (76.1%; n=191) followed by lack of accessibility (12.7%; n=32).  Over 11% (n=28) of the individuals were advised to wait for cataract surgery | NA | Among the ‘person-related’ barriers, the leading barrier was ‘lack of perceived need’ (61.1%; n=377) for reasons such as old age, good vision in the other eye, no one to accompany’ (20.3%; n=125), manageability of the problem (11.2%, n=97), not available/other priorities (3.4%, n=21), fear of cataract surgery (6.6%, n=41). | NA |
| 2 | Annual prevalence of unmet healthcare need in Thailand: evidence from national household surveys between 2011 and 2019 | Vuthiphan Vongmongkol, Shaheda Viriyathorn, Yaowaluk Wanwong, Waritta Wangbanjongkun and Viroj Tangcharoensathien, 2021 | Thailand | Analysis of eHealth and Welfare Survey (HWS) 2011–2019 | To assess the trend,between 2011 and 2019, of prevalence and reasons of unmet health care need and identifies population groups who had unmet needs. | The poor, the elderly  and people living in urban areas had higher unmet needs than their counterparts. | Coverage was available through primary health centers in all sub-districts and district hospitals in all districts nationwide. State Health Insurance is also available on a reimbursement basis to an extent. | Long waiting list. Non affordability of treatment cost. Non availability of beds for inpatient treatment. Lack of dental services in Living area | NA | Lack of time to seek care, inconvenient transportation, lack of knowledge about the available service provider availability, lack of companions to seek care, non-affordability of transportation cost, lack of information on availability of providers, lack of belief in health treatment. | The total unmet need in 2019  was high among elderly (4.8%) |
| 3 | Comparative health system performance in six middle-income countries: cross-sectional analysis using World Health Organization study of global ageing and health | Riyadh Alshamsan, John Tayu Lee, Sangeeta Rana, Hasan Areabi1 and Christopher Millett, 2017 | Six middle-income countries: China, Ghana, India, Mexico, Russia, and South Africa | Cross-sectional analysis from the World Health Organization Study on global Ageing and adult health, collected between 2007 and 2010. | To assess and compare health system performance across six middle-income countries that are strengthening their health systems in pursuit of universal health coverage | Cost remains a barrier to healthcare access in spite of insurance schemes. | NA | Less than 10% of respondents from India reported to have undergone free inpatient services in India. Health care deprivation is reported in India for vulnerable populations. Spending on medicines appeared to be a key factor in out-of-pocket spending | NA | NA | NA |
| 4 | Determinants of rural-urban differential in healthcare utilization among the elderly population in India | Shreya Banerjee, 2021 | Community setting- India. | Using the unit level data of Social Consumption: Health (Schedule number 25.0) of the 75th round of the National sample Survey conducted during July 2017–June 2018. | To the exploration of the prominent contributory factors behind the rural-urban gap in utilization of healthcare among the older population in India | Rural-urban disparities, educational and economic status are the crucial determinants on rural -urban difference in healthcare utilization | Health insurance coverage is available | Non-accessibility in rural areas, limited coverage in rural areas | Educational level, higher income of older adults, living arrangement (Never married, widowed, divorced or separated and older persons living alone had a lower rate of treatment seeking), economic independence among older people | Geographical division of place of residence (Rural elderly are less likely to utilize treatment services) | NA |
| 5 | Differentials in private and public healthcare service utilization in later life: do gender and marital status have any association? | Babul Hossain, K.S. James, Varsha P. Nagargoje & Papai Barman, 2021 | India | Based on randomized data collected through the 75th round of the National Sample Survey (NSS), corresponding to schedule number 25.0 (Social Consumption: Health), collected during July 2017-June 2018 (NSSO, 2019b). | To investigate whether the differentials in private and public inpatient healthcare utilization are associated with marital status for men and women aged 60 years and above in India. | Widowed men and women generally used public healthcare for hospitalization, while married men and women preferred private healthcare. Private inpatient health services expenditure was higher for married elderly than widowed elderly | NA | Unaffordability, less accessibility | Higher monthly income, education level, living arrangement (older adults with higher income, married and having better educational background prefer private health care), urban older adults prefer private care than rural old | Widowhood, living alone | NA |
| 6 | Equity and elderly health in India: reflections from 75th round National Sample Survey, 2017–18, amidst the COVID-19 pandemic | Alok Ranjan and V. R. Muraleedharan, 2020 | India | Analysis using: Data from the recently released (November 2019) 75th Round National Sample Survey (NSS), which was conducted from July 2017 to June 2018 | To highlight the vulnerability of the aged amidst the current COVID-19 pandemic, and in the light of the recent international evidence, suggests what government could do to mitigate their vulnerability | Hospitalization rate was higher in the urban areas (OR: 1.23), general social category (OR: 1.18), richest economic quintile (OR: 1.69), and among those living alone (OR: 2.40)., Among the elderly, 64% of those in the scheduled tribe (social group) and 51% in the poorest economic quintile utilized public facilities for hospitalization | Publicly funded health insurance (PFHI) coverage and (tax funded) subsidized public provisioning is available | Proximity to the place of residence | NA | Economic dependency of aged. | Public healthcare utilization was higher in rural areas (39.7%), ST category (43.5%), casual laborer (50.3%), illiterate (37.7%), never married or divorced (47.6%) and poorest economic quintile compared to their respective counterparts (rural-45.3%, urban-41.6% |
| 7 | Examining the predictors of healthcare facility choice for outpatient care among older adults in India using Andersen’s revised healthcare utilization framework model | Margubur Rahaman, Pradip Chouhan, Avijit Roy, Md. Juel Rana and Kailash Chandra Das, 2022 | India | Secondary analysis using the first wave of the longitudinal Ageing Study in India (LASI, 2017–18). | To examine the level, patterns, and factors associated with outpatient care use. | Years of schooling, household wealth status, place of residence, self-rated health, and health insurance were all found to be significant determinants of public or private facility use. | Health insurance, closeness to care facilities, home visits, health camp, availability of pharmacy /drug store, accessibility to NGO/charity hospitals/Private Ayush hospitals, Private nursing homes/OPS (Private facilities), accessibility to PHC/Sub centers/CHC etc.(Public care) | NA | Wealth status, Better education | Illiteracy | NA |

| 8 | Factors associated with access to palliative home care in palliative patients at Lampang Hospital | Weerakorn Yongja, Phornwipa Panta, Napat Phetkub, Wararat Thatayu, Win Techakehakij, 2022 | Thailand | Retrospective study using data from electronic medical records at Lampang Hospital | This study explores  factors associated with successful delivery of palliative home care in palliative patients. | Being female, having low palliative performance scale score, morphine use, and having Civil Servant Medical Benefit Scheme insurance are associated with a higher chance of having access to palliative  home care, compared with their counterparts | NA | NA | The majority of samples (81.6%) were covered by the universal coverage insurance | NA | Patients under the Civil Servant Medical Benefit  Scheme insurance had 2.052 times higher odds of receiving  PHC, compared with those under Universal Coverage  insurance |
| --- | --- | --- | --- | --- | --- | --- | --- | --- | --- | --- | --- |
| 9 | Factors Associated with National Health Insurance Coverage in Indonesia. | Tintin Sukartini, Hidayat Arifin, Yulia Kurniawati, Rifky Octavia Pradipta, Nursalam Nursalam, Joel Rey Ugsang Acob, 2021 | Indonesia | Retrospective cross-sectional data  Demographic and Health Survey 2017 | This study aims to analyze the factors associated with health insurance coverage in Indonesia. | The prevalence of health insurance coverage in the Indonesian population is 62.3%. Respondent aged 15-24 years, secondary education level, poorer wealth index, live in rural area, divorced were less likelihood to have health insurance. Conversely, the respondent who received earnings was more likely to have health insurance. | NA | NA | The multivariate analysis shows that age, education level, wealth quintile, marital status, and earnings have very significant  relationships with health insurance coverage. | NA | The majority of respondents with health insurance were in the age range of 35–49 years (51.05%) |
| 10 | Factors associated with psychiatric disorders and treatment seeking behavior among older adults in India. | Shobhit Srivastav, KM Sulaiman, Drishti Drishti & T. Muhammad, 2021 | India | Analysis using LASI data  60 years and above | This study investigates the associated factors of psychiatric disorders and its treatment seeking among older adults in India | About 2.8% of older adults had psychiatric disorder and of those who were suffering from psychiatric disorder, 41.3% (out of 2.8%) sought medical treatment. It was found that older adults who ever worked but currently not working, who had low level of life satisfaction, had poor self-rated health, had difficulty in activities of daily living (ADL) and instrumental ADL and had symptoms of psychological distress had higher probability of suffering from psychiatric disorder in reference to their counter parts | NA | NA | Older adults who had higher and above educational status was seeking treatment for psychiatric disorder, older adults seeking treatment for psychiatric disorder was higher among separated/never married/divorced, Higher percentage of older adults from urban place of residence were seeking treatment for psychiatric disorder | The chances of older adults who lived in remote areas from the health facility were 0.82 times significantly less likely to seek treatment for psychiatric disorder in comparison to their counterparts | Older adults from oldest-old age group, who were females, from poorest wealth quintile, from Scheduled Tribe and from eastern region had lower probability of seeking treatment for psychiatric disorder in reference to their counterparts, male older adults seeking treatment for psychiatric disorders is more. |
| 11 | Factors associated with the use of outpatient services among the elderly in Indonesia. | Ema Madyaningrum, Ying-Chih Chuang and Kun-Yang Chuan, 2018 | Indonesia | Cross sectional study using data from the Indonesian Family Life Survey 5 (IFLS 5) | Hence, this study aimed to identify factors related to the use of outpatient services among the Indonesian elderly. | Factors associated with the use of outpatient services were socioeconomic status, insurance status, regions of residence, self-rated health, and the number of chronic conditions. | NA | Disparities were probably due to differences in healthcare coverage, in the availability of service providers. | Those with health insurance were 1.38 (95% CI: 1.14–1.67) times more likely than their counterparts to use outpatient services | NA | In general, being female, higher economic status, worse health, and urban regions were associated with the use of outpatient services, more chronic conditions was also associated with higher use of outpatient services. |
| 12 | Factors Influencing Access to Health Services among Chronically Ill Older Adults with Physical Disabilities in the Era of the COVID-19 Outbreak. | Sutham Nanthamongkolchai, Athicha Tojeen, Korravarn Yodmai and Wanich Suksata. 2023 | Thailand | Cross-sectional study | This study aimed to examine the association between social support, perception of benefits due to disability and access to health services among chronically ill older adults with physical disabilities during Covid 19 crisis in Thailand | Most participants perceived benefits (58.8%) and access to health services (56.2%) at good levels, while was at a moderate level (47.9%), t social support (β = 0.351), perception of benefits (β = 0.257) and age (β = 0.167) were positively correlated with health service access. | NA | NA | NA | Lack of social support | The overall access to health services of the participants was at a good level (56.20%, n = 150), social support, perception of benefits and age (β = 0.351, 0.257, and 0.167, respectively); these factors predicted the participants’ access to health services with an accuracy of 23.3% |
| 13 | Health Care Utilization by Older Adults in Nepal: An Investigation of Correlates and Equity in Utilization | Saruna Ghimire, Devendra Raj Singh, Sara J. McLaughlin, Renusha Maharjan and Dhirendra Nath, 2022 | Nepal | Multivariable logistic regression analyses | Study aims to evaluate factors associated with health care utilization (HCU) and to assess vertical and horizontal equity in utilization among Nepali older adults. | Study demonstrates marked horizontal inequities in health care, with utilization a luxury afforded to older citizens with high income, general lack of awareness of existing no-cost health services. | Governments’ provision of free primary health services and treatment subsidies for severe health conditions for older adults | Lack of awareness of the services, Lack of transportation, geographically distant health facility, the cost associated with transportation and lodging, dissatisfaction with health care quality. | Living in a nuclear family, having children attend health care visits, people with greater income increased the odds of health care use. | Preference for traditional medicine discourages use of health services even when free of charge. Poor social networks and social support are seen as a barrier to access. | Presence of chronic diseases is associated with greater health care use This suggests that the principle of vertical equity. |
| 14 | Health needs, access to healthcare, and perceptions of ageing in an urbanizing community in India: a qualitative study | Nandita Bhan, Pavitra Madhira, Arundati Muralidharan, Bharati Kulkarni, GVS Murthy, Sanjay Basu6 and Sanjay Kinra, 2017 | India | Qualitative study | To understand perceptions of health needs of the elderly across three key local stakeholder groups (community, health providers and administrators) in an urbanizing rural area | All stakeholders agreed that compared to government health services, private providers were available when needed, provided diagnostic support and, in general, better treatment. | Health service needs were addressed by mobile health providers (104 mobile health service) | Access to diagnostic services was a challenge for the elderly due to their frailty, limited mobility and dependence on family members for transport, Patient dissatisfaction due to lack of systems for accountability and monitoring. Poverty was a major barrier in accessing nutritious food and medicines. | NA | Community reported neglect and abuse as key household issues influencing health care use, caregiving, and general wellbeing among the elderly. Limited household resources and changing cultural values were considered as reasons for neglect of the elderly within households, Intergenerational disagreements and changing obligations towards older members often led to neglect of health issues of the elderly. | NA |
| 15 | Health service use, out-of-pocket payments, and catastrophic health expenditure among older people in India: The WHO Study on global AGEing and adult health (SAGE) | Ethel Mary Brinda, Paul Kowal, Jørn Attermann, Ulrika Enemark,2015 | India | Quantitative | Aimed to study the determinants of health service use, out-of-pocket and catastrophic health expenditures among older people in one LMIC, India. | Out-of-pocket health expenditures were higher among participants with disabilities and lower income. | Free or low-cost public healthcare services like Aarogyasri and Rastriya Swasthya Bima Yojana programs | The study identiﬁed lower rates of healthcare utilization among older people with high needs. Persistent need for high out-of-pocket health expenditure may force disabled, poor, older people to either forego or underuse healthcare. | NA | NA | NA |
| 16 | Health Services Utilization Barriers for Rural Elderly Women in Bangladesh: Narratives of Clinicians, Pharmacists and Public Health Assistants | Mohammad Hamiduzzaman1 · Anita De Bellis, Wendy Abigail, Evdokia Kalaitsidis, 2022 | Bangladesh | Qualitative study | Aims to explore discrimination and marginalization that determines women’s access to healthcare from the staff perspective. | Unequal distribution of health services; marginalization in patient-staff relationships; living with poverty; social relegation; and mistrust of clinical treatment. | NA | Health system focus largely on maternal care and hence current policies and programs discouraged the staff in recognizing other healthcare needs of the women. A lack of interest among staff, inadequate number of public healthcare facilities and lack of facilities in the health centers, shortage in the staff number, no geriatric specialized services, in combination with the lack of availability of vehicles, further impacted on the women not accessing healthcare. | NA | This lack of communication because of cultural and traditional practices creates an inability of communication that also combines with power relationships in a patriarchal society, lack of financial capacity, dependency of rural elderly women. | NA |
| 17 | Horizontal Inequity in Elderly Health Care Utilization: Evidence from India | William Joe, Shalini Rudra and S V Subramanian, 2015 | India | Cross sectional survey | Aims to explore the horizontal inequity in Elderly Health Care Utilization | NA | Coverage of insured population may have increased due to several national (such as Rashtriya Swasthya Bima Yojana) and subnational policy initiatives (such as Arogyashree Scheme). | Income-related inequities and inequalities in health care utilization are a prominent concern for elderly well-being. | NA | Horizontal inequality | NA |
| 18 | Implication of Adult Out‑Migration on the Health and Healthcare Seeking of the Older Parents in Indian Households: An Exploration | Pravat Bhandari, Shinjini Ray, R. Nagarajan, 2021 | India | Cross-sectional survey | Study investigates the impact of adult out-migration on various health dimensions of left-behind older parents and their treatment seeking behavior in Indian context | Study provides evidence that absence of adult children due to migration has both positive and negative health impacts on the older parents left behind. | NA | Older individuals with a greater number of migrant children who suffered from chronic disease were less likely to receive medical and other care facilities., | Noted that the migration of adult children increases the probability of being diagnosed with chronic morbidity among the older parents left-behind, migration has a direct and positive influence on older individuals’ functional ability to perform the instrumental activities. | NA | NA |
| 19 | Older adults with non-communicable chronic conditions and their health care access amid COVID-19 pandemic in Bangladesh: Findings from a cross-sectional study | Sabuj Kanti Mistry, A. R. M. Mehrab Ali, Uday Narayan Yadav, Saruna Ghimire, Md. Belal Hossain, Suvasish Das Shuvo, Manika Saha, Sneha Sarwar, Md. Mohibur Hossain Nirob, Varalakshmi Chandra Sekaran, Mark F. Harris,  2021 | Bangladesh | Cross-sectional design | Aimed at exploring the challenges experienced by older Bangladeshi adults with non-communicable chronic conditions  in receiving regular health care services during the COVID-19 pandemic | Participants with at least one condition (AOR: 1.95, 95% CI: 1.33–2.85) and with multi morbidity  (AOR: 4.75, 95% CI: 3.17–7.10) had a higher likelihood of experiencing difficulties accessing medicine. | NA | About one in four participants experienced difficulties accessing medicine (23%) and receiving routine medical care, access to health care for non-communicable chronic conditions is an ongoing issue and existed in Bangladeshi society. Distance to healthcare facilities and transportation, out-of-pocket medical expenses are, many of the tertiary care centers were converted into COVID-19 hospitals, limiting access to regular health care services. | In Bangladeshi society, the male adult is morally obliged to provide care and support to their older parents. | NA | NA |
| 20 | Older adults with pre-existing noncommunicable conditions and their healthcare access amid COVID-19 pandemic: a cross-sectional study in eastern Nepal | Saruna Ghimire, Aman Shrestha, Uday Narayan Yadav, Sabuj Kanti Mistry, Bunsi Chapadia, Om Prakash Yadav, ARM Mehrab Ali, Lal B Rawal, Priyanka Yadav, Suresh Mehata, Mark Harris, 2022 | Nepal | Cross sectional survey | Study investigated access to healthcare by Nepali older adults with pre-existing conditions during the COVID-19 pandemic. | Older adults with pre-existing conditions in Nepal, who require routine medical care and medication, faced significant difficulties obtaining them during the pandemic. | NA | Those with multiple conditions were even more likely to experience difficulty accessing healthcare than those with single conditions despite their likely increased need, inaccessible healthcare is a long-term problem in Nepal, existent even prior to the COVID-19 pandemic, the health system may not have been able to maintain essential services because of limited human resources for health. | NA | NA | NA |
| 21 | Relying on Whom? Correlates of Out-of-Pocket Health Expenditure among the Rural Elderly in Odisha, India. | Pradhan, J, Dwivedi, R. & Banjare, P., 2017 | India | Cross sectional | To assess the healthcare utilization pattern among rural elderly and second, to examine the various covariates of Out-of-Pocket Expenditure (OOPE) in Odisha, India | A major source of financing for the elderly population was their son for both inpatient and outpatient healthcare in the last episode of illness. | More than half of the population was availing care from the government providers, followed by others (i.e. Ayush, NGO, and Charitable institutions). Reasons for preferring public healthcare providers over private and others may be financial or availability of the facilities in the rural areas. | Source of finance for the private sector (out of pocket expenses) | Major source of financing for inpatient care among the elderly population was their son followed by, self-financing, their spouse and son in law/daughter in law in the last episode of illness. | NA | NA |
| 22 | Self-reported Seasonal Symptoms and Diseases and Primary Healthcare Utilization Among Rural Elderly Women in Sylhet District, Bangladesh | Mohammad Hamiduzzaman, 2020 | Bangladesh | Mixed-methods approach | This study aims to identify the health effects of seasonal variations that place increased risk of symptoms and diseases on rural elderly women, and to explore the determinants associated with the women’s use of healthcare locally. | Utilization of primary healthcare was low, and marginalization in using healthcare was underpinned by the health system, the poor living conditions of the women, and their reluctance to seek treatment, age, saving or bank balance, risk behavior (smoking), non-functionality, hearing defect and multi-morbidity are significantly influencing the spending pattern of the elderly on healthcare. | NA | Shortage of doctors, nurses and health assistants, absence of clinicians, ( one medically trained doctor practicing in union clinics for one day per week) , lack of supply of equipment and medications resulted in a lack of care support for rural elderly women , no geriatric service in the union clinics, pregnant woman and children service were more , lack of geriatric education and training to healthcare staffs , lengthy waiting period , poor clinical interactions resulted in a delayed and inappropriate access, lack of communication, verbal abuse and bad behaviors of the staff. | NA | Lack of personal income/savings and financial support from family members and the government, staff indicated poor living conditions among the rural elderly women (REW) because of a lack of engagement in the formal employment sector, number of REW receiving the elderly allowance was low, lack of health literacy among the women, believing in superstitions, tendency to rely on religion and self-medication | The low rate of health literacy among the women was identified as a determinant having an impact on the REWʼs use of local clinical treatments in a timely manner as well as the women’s health beliefs. |
| 23 | The World Is Not Mine, Barriers to Healthcare Access for Bangladeshi Rural Elderly Women | Mohammad Hamiduzzaman & Anita De Bellis & Wendy Abigail & Evdokia Kalaitzidis & Ann Harrington, 2021 | Bangladesh | Qualitative | This study aimed to explore how this cohort related their healthcare access to their living circumstances and provided insight into how their healthcare access needs can be addressed. | Study confirms that the rural elderly women require adequate policy responses from the government and need multiple support systems to secure adequate access to healthcare. | NA | Lack of services, travel difficulties, a shortage of doctors, scarcity of medical resources, extensive waiting periods, costs of care and maltreatment by healthcare providers. There was a lack of medical equipment and medications that contributed to a delay in their access to diagnosis and treatments. | NA | Lack of education and low level of health literacy, inability to comprehend health information, healthcare beliefs in traditional religious healing, poor household income, lack of or poor financial support from the government resulting in poverty and so lack of accessibility, social isolation, women revealed they had responsibility to consider the healthcare needs of the young and the male family members before their own needs. | A fear of creating inconvenience for others was identified which prevented these women from seeking, Self-care included praying, self-treatment, and the use of traditional healing. |
| 24 | Ageing in India: Financial hardship from health expenditures | Ting‐Hsuan J. Lee, Indrani Saran, Krishna D. Rao,  2017 | India | Using NSSO survey | Investigate the association between age and financial hardship due to health expenditures. | Individuals aged 60 and above had a much higher probability of becoming impoverished as a result of health expenditures. | NA | Mean annual out‐of‐pocket health expenditure of  Rs. 7573 for older individuals was more than twice that of an average individual. A total of 59 million households experienced catastrophic health expenditures, 23 million (39%) of which were households with members aged 60 and above. Approximately 1.7% of men under the age of 60 experienced impoverishment due to health expenditures in 2014. The probability of experiencing impoverishment due to health expenditures was approximately 3 percentage points higher for individuals aged 60. | NA | NA | NA |
| 25 | Exploring the System Determinants Associated with Senior Women’s Access to Medical Care in Rural Bangladesh | Mohammad Hamiduzzaman, Anita De Bellis, Wendy Abigail, 2022 | Bangladesh | Qualitative exploration | Study aims to explore the system determinants that impact on rural senior  women’s utilization of healthcare. | Three major themes emerged from the system determinants: legal framework of aged care; inadequate healthcare support; and professional knowledge and skills of healthcare staff that led the rural senior women to avoid or delay access to hospitals and clinics. | NA | Senior women receiving little attention from policy makers or health system authorities in upholding their right to healthcare access, some healthcare staff lacked interest in establishing easier access for the women. The participants identified poor access for the women, lack of and unavailability of healthcare services, the high cost of medical care, low staffing levels, an inadequate supply of equipment and medication, the long waiting times for treatment and an inadequate referral system. Most of the staff entered the healthcare sector without adequate or formal geriatric education and/or training; and (b) the medical education or training received by the staff was related to maternal and child health. | This legal framework involved a combination of constitutional rights, healthcare policies and programs and the protection of human rights in clinical settings. | Ineffectiveness of constitutional laws in meeting healthcare needs of older adults, women in particular, lack of knowledge among the women regarding their constitutional and legal rights. | NA |
| 26 | Exploring the road to public healthcare accessibility: a qualitative study to understand healthcare utilization among hard-to-reach groups in Kerala, India | Surya Surendran Jaison Joseph, Hari Sankar, Gloria Benny and Devaki Nambiar, 2024 | Kerala, India | Qualitative | To ascertain the initial impacts of these measures among ‘hard to reach groups’ as part of a larger health policy and systems research study, with a focus on public sector health service utilization | Hard-to-reach groups, including older adults in Kerala, face several challenges in health care utilisation; interrelated barriers—such as physical inaccessibility, limited services at lower-level facilities, information gaps, and opportunity costs—which hinder equitable access to public healthcare | The presence of public insurance for inpatient care in government hospitals increases utilisation among poorer and vulnerable groups. Primary Health Centres (PHCs) and Family Health Centres (FHCs) cover a range of essential services, which benefit those with chronic illnesses needing continuous care. Some groups, such as tribal communities and the elderly, receive preferential treatment in public facilities, allowing for faster healthcare access. | #Limited Availability at Lower Levels: Public primary centers (FHCs, sub-centers) often have limited services and doctors #Long Waiting Times and Delays: Overcrowding at higher-level public facilities leads to long queues, #Inadequate Medicines and Diagnostics: Many medicines and diagnostic services are unavailable at public facilities #Lower Quality of Care: Lower-level facilities are often perceived as less capable #Information Inaccessibility: Many beneficiaries lack adequate information on public insurance | #Proximity (In some areas): Where public or higher-level facilities are close to home or easily accessible #Community Ties and Information: Stronger links to local government or NGOs can increase awareness and utilisation of public services. | NA | NA |
| 27 | Morbidity, Treatment-Seeking Behaviour, and Out-of-Pocket Expenditures Among the Tribal Geriatric Population: A Cross-Sectional Study From a South Indian District | Ashish Solanki , Godavarti D. Kumar , Godi R. Varma, Bontha V. Babu, Yadlapalli S. Kusuma, 2025 | India | Cross- sectional | (i) To examine morbidity patterns, (ii) to explore the treatment seeking behaviours, and (iii) to analyses the out-of-pocket expenditures (OOPEs) among Indigenous (referred  to as tribal in India) elderly in the Visakhapatnam district of Andhra Pradesh, India. | Out-of-pocket expenditures (OOPE) were substantially higher for private healthcare compared to government services, with hospitalisation costs far exceeding monthly incomes, leading some elderly to borrow money or sell assets.  Systemic barriers included poor infrastructure, distance to facilities, limited diagnostics, and transport challenges; non-systemic barriers involved poverty, low health literacy, and cultural factors. | A significant proportion of geriatric patients relied on government health services for acute (81.6%), chronic (42%), and hospitalisation (70.6%) needs Government Support Schemes: Old age pensions Mahatma Gandhi National Rural Employment Guarantee Act, and state agricultural support contributed to at least some financial security. Expansion of Ayushman Bharat: Conversion of sub-health centres into Health and Wellness Centres (HWCs) under Ayushman Bharat #Community Health Workers: The presence of trained health workers (like ASHAs and ANMs) who regularly visit villages improves basic healthcare accessibility and health-seeking behaviour. | #Poor Infrastructure | #Community and Family Support: financial, social, and emotional support,   #Traditional Knowledge & Resilience: Elders possess cultural and traditional health knowledge, which sometimes translates into self-management or preventive practices. | Poor road connectivity and lack of public transport.  Socioeconomic Poverty: 69.6% have an income below INR 5,000 per month, and many rely on government pensions.  Limited Health Awareness and Literacy Cultural and Social Barriers: Belief in traditional healers, lack of trust in formal systems, and language/cultural dissonance  Psychosocial Stressors: Widowhood, lack of companionship, low income, and minimal intergenerational support can exacerbate both physical and mental health challenges for older adults | NA |
| 28 | Health facility utilisation and Healthcare‑seeking behaviour of the elderly population in India | Roopani, Neha Dumka, Tarannum Ahmad, Erin Hannah, Atul Kotwal, 2023 | India | Cross-sectional study Longitudinal Ageing Study in India (LASI)‑Wave I | To provide the status of health facility utilisation, health‑seeking behaviour (HSB),  and factors influencing them. | Enhancing health insurance coverage, specialized infrastructural investment, and targeted social interventions (education and outreach programs) are likely to substantially improve elderly healthcare utilization and health-seeking behaviour in India. | Government Programs: The National Programme for Health Care of the Elderly (NPHCE) and Ayushman Bharat Health and Wellness Centres (HWCs)  Health Insurance Initiatives:  #Expanded Service Packages: HWCs and tertiary centres. | #Limited Public Healthcare Infrastructure  #Shortage of Specialized Medical Practitioners  #Low Public Investment:  #Insurance Coverage Gaps  #Lack of Comprehensive Elderly Care models | # Higher Education and Income #Urban residence  #Marital status | #Geographical Disparities.  #Low Education Levels  #Economic Constraints of those belonging to the lower MPCE quintiles (monthly per capita expenditure)  #Female elderly report lower utilisation of inpatient services, # low awareness and Health Literacy  #widowhood and Social Support: | NA |
| 29 | Factors affecting access to health services by older adults in an urban community in Thailand: a cross-sectional study | Areeya Jirathananuwat, 2023 | Bangkok, Thailand | Cross-sectional survey | To study factors affecting access to health services utilization of older adults in urban communities in Thailand | Using health insurance and (OR=1.94, p=0.006), (2) perceived necessity of care/health awareness (OR=1.54, p=0.043) affect healthcare utilisation | Universal Coverage Scheme (UCS).  Health insurance rights  Standardised healthcare costs | # Insurance is restricted only to one place where it is registered # lack of options  Limited human resources  # long waiting times for doctors. | #Health awareness  #Use of various transport options to reach the hospital | # Proximity of public health facilities  #low income  #lack of insurance for certain needs beyond coverage |  |
| 30 | Social health insurance, family support,  and chronic diseases as determinants  of health service utilization among senior  citizens in rural Nepal | Poudel et al, 2025 | Nepal | Community-based cross-sectional study | To assess the status of health service utilization and its influencing factors among senior citizens in rural municipalities of the Kaski district, Nepal. | 54.6% of senior citizens visited healthcare facilities in the past 12 months.  Utilization was significantly associated with:  Awareness of free healthcare services  Membership in social health insurance  Family support  Self-perceived health status  Independence in activities of daily living  Having a chronic disease | Social health insurance (SHI) membership: Strongly increases utilization.  Awareness about free healthcare services: Those aware are more likely to use services.  Government policies: Provision of free and subsidized healthcare for senior citizens, with free premiums and increased coverage. | Implementation gaps and insufficient legal frameworks that hinder support for the elderly.  Inadequate drugs or equipment in health facilities (reported as a reason for non-utilization).  Lack of knowledge of healthcare facility locations and services for some seniors. | Family support: Seniors from highly functional families are more likely to utilize healthcare.  Social protection programs that provide indirect support for health-seeking behavior. | Lack of family support: Seniors from dysfunctional families less likely to utilize care.  Transportation/barrier costs: Cost and lack of transport prevent access.  Agricultural-based low income and lack of social support in rural areas. | Main reasons for non-utilization: Perceived lack of illness (55.1%), transportation cost (23.6%), not knowing where to go (12.4%).  The presence of multiple chronic diseases strongly predicts service utilization.  Seniors with independence in daily living activities actually showed lower utilization, possibly reflecting that sicker (less independent) seniors needed more care.  No significant association was found between economic status, distance to health facility, education level, or sex and utilization in this study. |
| 31 | Understanding inequalities in spatial accessibility to multi-tier healthcare for older adults in rapidly aging Bangladesh | Ahmed et. al, 2025 | Bangladesh | Spatial accessibility analysis using the Enhanced Two-Step Floating Catchment Area (E2SFCA) method and Gini coefficient inequalities, utilizing geocoded health facility data, road networks, and population statistics for older adults (age 60+). | To assess spatial accessibility to primary, secondary, and tertiary public healthcare for older adults in Bangladesh.  To quantify inequalities in access across tiers and space using the Gini coefficient.  To identify geographic disparities and provide evidence for targeted interventions. | #Healthcare accessibility in Bangladesh is unequally distributed spatially and across care tiers.  #Primary care: generally accessible, but with pockets of poor access in rural and remote regions.  #Secondary care: less evenly distributed, concentrated more in urban centers, resulting in regional disparities.  #Tertiary care: most unequally distributed and concentrated in major cities, with the majority of rural and remote populations having very poor access.  Inequality (Gini coefficients) in accessibility increases from primary to tertiary care.  Tertiary care shows the highest levels of inequality in every division but Dhaka and Chattogram.  No division achieved both high accessibility and low inequality for any tier.  #Travel time is significant:  #Urban-rural disparities:  Urban areas have better facility concentration but experience demand overload.  Rural regions face “service deserts” especially for higher-tier care. | Relatively widespread primary care infrastructure: More evenly dispersed compared to higher care tiers, reduces some spatial gaps.  Government commitment to low-cost public services: For poor and older populations, primary public facilities are the main access point. | Severe urban bias at higher tiers: Most secondary and nearly all tertiary services are urban-centric, leaving rural elderly at a disadvantage.  Resource limitations and inefficiencies: Shortage of hospital beds, lack of specialized providers in rural areas, and highly centralized tertiary care.  Overwhelming demand in urban areas: Even with more facilities, urban services face overload, causing long waiting times and logistical challenges for older adults | Availability of informal and low-cost transport options: Rickshaws and auto-rickshaws (CNGs) serve as main modes for many, offering localized mobility for primary care.  Community clinics (excluded from final analysis due to limited services): Still relevant for the most basic and preventive services at the local level. | Inadequate, fragmented, and unreliable transportation: Especially problematic for rural elderly long travel times, poor public transport, and reliance on physically demanding modes.  Economic constraints: Many older adults lack health insurance and require out-of-pocket payments. Cost of travel and treatment deter help-seeking.  Social and mobility challenges: Older people with poor health or insufficient caregivers are disproportionately affected, often foregoing needed care due to travel or logistical burdens. | Decentralize secondary and tertiary services to underserved regions.  Invest in age-friendly, affordable public and paratransit transport.  Integrate healthcare and mobility planning in policy.  Identify and address specific “lacking service zones” for priority investments. |
| 32 | Determinants of accessing healthcare services for outpatient care: A study on older adults in India | Rahaman et. Al, 2024 | India | Cross sectional analysis using LASI | To examine determinants influencing older adults’ choice between public, private, and other outpatient healthcare services | Private services were the most used (57.3%), followed by public (30.2%) and other services (12.4%). | Presence of national and state health insurance schemes (e.g., ABPM-JAY, RSBY) that can reduce financial barriers.  Public facilities provide more affordable care compared to private, important for poorer households. | #Inadequate infrastructure, shortage of skilled staff, poor quality of care in public sector → underutilisation.  #Limited insurance coverage for outpatient care services.  #Long waiting times, lack of privacy, poor cleanliness in public facilities.  #High out-of-pocket costs for private services causing financial strain. | #Higher education improves awareness and health literacy, increasing ability to navigate health systems.  #Wealth status enhances ability to afford both public and private services. | #Socio-economic inequality -poor and illiterate older adults often rely on expensive private services due to lack of quality public care, risking impoverishment  #Rural-urban disparity in service availability and accessibility.  Gender and social status can influence service choice and access. | #Pro-rich bias: Wealthier older adults benefit more from both public and private healthcare utilisation.  #Significant portion of the poorest still depend on private care, increasing financial vulnerability.  #Policy needs: Strengthen public sector infrastructure, improve consultation experience, expand insurance coverage (especially to BPL elderly), and ensure quality in non-clinical aspects to move toward Universal Health Coverage. |
| 33 | India’s immobile elderly and universal health coverage: insights from 75th round national sample survey | Ranjan et. Al, 2024 | India | Quantitative | #To explore disease burden and utilisation of healthcare services by immobile elderly.  #To evaluate financial protection (OOPE, CHE, insurance coverage).  #To inform policy for universal health coverage (UHC) with focus on immobile elderly. | #Prevalence: 7.5% elderly immobile — 1.4% bedridden, 5.5% confined to home, 0.6% in wheelchair.  Unmet healthcare needs higher in immobile elderly, especially rural, poorest, illiterate, and widowed groups.  #Borrowing to finance hospitalization common.  Insurance: Overall elderly coverage = 18.9%; PFHIs dominate but mostly for inpatient care only — outpatient and rehabilitative excluded. | Public healthcare more affordable & more used by poorest elderly.  PFHIs (e.g., PM-JAY) provide financial risk protection for hospitalisation. National Health Policy 2017 recognizes need for rehabilitative & palliative care. | #Severe shortage of rehabilitative services & allied health professionals in public sector.  #Private sector dominant but expensive high OOPE, high CHE.  #Insurance schemes exclude outpatient & rehabilitative services (most needed by immobile elderly).  Long travel distances, poor infrastructure, lack of specialist services in rural public facilities. | #Better education reduces odds of immobility and improves disease management.  #Higher economic status enables access to both public and private care.  #Having family support (married, children) reduces isolation and unmet care needs. | #Elderly in poor, rural, illiterate, or marginalised groups face double disadvantage (higher immobility + lower access).  Increasing migration of younger family members leaves elderly isolated.  Economic dependence on family-prioritisation of other needs over elderly healthcare. | #Psychiatric and neurological disorders form a vicious cycle with immobility.  #Even poorest elderly often use private services due to public sector gaps. |
| 34 | Elderly Health in Different States of India: Learnings from 75th Round National Sample Survey, 2017–2018 | Alok Ranjan & Prithivi Prakash Sivaprakash, 2025 | India | Quantitative | #To examine elderly health status across states at different ETLs.  #To measure healthcare utilisation (outpatient & inpatient) and unmet healthcare needs.  #To assess financial risk protection (OOPE, CHE, borrowing) for elderly.  #To explore living arrangements, economic dependence, physical immobility, and self‑perception of health. | #Unmet needs: National average 8.1%; very high in Meghalaya (58%) & Bihar (44.6%), low in Kerala (3.9%) & Tamil Nadu (4.1%).  #Higher unmet needs for STs, illiterate, poorest quintile, uninsured, casual labour households, elderly living alone. #Financial protection:Overall insurance coverage = 18.9% (as high as 76.2% in Andhra Pradesh, as low as <2% in many states).  #Living arrangements: 4.2% live alone; highest in Andhra Pradesh (10.3%) & Tamil Nadu (8.4%).  #Economic dependence: 70% partially/totally dependent on others. | Southern & northeastern states show higher public sector usage due to better availability and investment.  PFHIs (e.g., PM‑JAY) exist for inpatient care in several states.  Some UTs have high public hospital bed capacity per capita. | #Private sector predominance high OOPE & CHE.  #Limited geriatric, chronic disease, and rehabilitative care in public facilities.  #Low overall insurance coverage; outpatient care not covered.  #Weak public health systems in low ETL states. | #Higher ETL states also have higher life expectancy & better disease detection.  #Stronger education and awareness linked to lower unmet needs.  #Social support from living with family reduces borrowing/unmet needs. | #Economic dependence prevalent among elderly; poverty worsens access.  #Living alone linked to higher unmet needs & borrowing risk.  #Social isolation, especially in high ETL states, increases vulnerability. | #Health disparities across states largely align with ETL differences: high ETL = higher NCD burden, more health service use, lower unmet needs, but also higher proportion immobile/elderly living alone.  #Public sector needs realignment toward chronic and elderly care. |
| 35 | Are Informal Older Workers Utilizing Less Healthcare Services? Evidence from the Longitudinal Ageing Study in India, Wave-1 | Poulomi Chowdhury & Akansha Singh, 2024 | India | Quantitative, cross-sectional analysis | #To examine whether healthcare utilization differs between informal and formal older workers in India  #To assess how this relationship is influenced by chronic health conditions (CHC), depression, and socio-economic factors  #To identify determinants of zero healthcare visits among older workers | #Overall, informal workers have fewer visits than formal workers (IRR=0.958, p<0.001).  #Formal workers with CHC and/or depression have higher visits; informal workers with same conditions have significantly fewer visits:  #Depression: 0.892× (p<0.05) fewer visits than formal equivalents  #CHC+Depression: 0.847× (p<0.0001) fewer visits than formal equivalents  #Informal workers without health problems had more visits than formal workers without problems — possibly due to physically demanding work causing other acute health issues. | Public healthcare aims to provide equitable access, especially for vulnerable workers  National health insurance schemes (e.g., Ayushman Bharat) exist, though coverage is limited for elderly  Urban areas offer greater provider choice | #Pro-rich bias in utilisation, with poorer & informal workers under-utilising despite need  #Public facilities: inadequate infrastructure, long waiting times, poor quality - deters use  #Insurance coverage gaps: limited awareness, outpatient exclusions, high out-of-pocket costs even for insured  #Rural-urban disparity in facility access | #Higher wealth & education linked to greater healthcare use  #Physically demanding informal work may prompt care-seeking for injuries/pain  #Cultural & family support can facilitate seeking care | #Economic vulnerability reduces treatment-seeking, especially for costly NCDs  #Informal workers risk income loss if time taken off for treatment  #Social disadvantage (tribal, rural, poor) linked to lower utilisation  #Low awareness of available schemes & services | #Informal older workers with serious health conditions are disproportionately under-utilising healthcare — worsening health vulnerability  #Those without insurance or from marginalised communities are at highest risk of zero healthcare visits |
| 36 | Demography Integrated Surveillance for Health Assessment‑Geriatric (DISHA‑G), a rural cohort of older adults in Odisha, India | Jaya Singh Kshatri et al, 2025 | India | Longitudinal rural cohort | #To build a first-of-its-kind geriatric community cohort in rural eastern India  #To profile socio-demographic & health characteristics of older rural adults  #To examine healthcare-seeking preferences & determinants of informal care use  #To provide a longitudinal platform for studying ageing, multimorbidity, and care access | #Demographics: 52.7% male; most aged 50–59 (44.8%); older ages predominantly female  #Education: 26.9% no formal education; 12.1% had graduation or above  #Occupation: 53.8% self-employed (including agriculture); 20.5% homemakers; 13.3% daily wage earners  #Access: 83% had internet; 63% had safe drinking water; 45.6% had health insurance (96.9% govt schemes) | Public facilities geographically accessible and low-cost for most villagers  Presence of government-provided health insurance schemes (e.g., AB-PMJAY-type)  Functioning primary and secondary public facilities in district with relatively good reach | #Limited private sector availability in rural setting—yet not always matched by strong public services  #Informal care reliance indicates service gaps in availability, quality, or awareness  #Underdiagnosis of chronic conditions due to limited screening and health literacy  #Insurance coverage gaps (e.g., outpatient care, diagnostics)  #Potential long wait times and limited specialist availability in public sector | #High family cohesion (nuclear & extended families) as potential support base  #Community volunteer network supports enrolment, mapping, and health awareness  #Government welfare linkages present in the villages | #Lower wealth reduces likelihood of formal care use  #Social norms & low perceived need suppress preventive care-seeking  #Digital/infrastructure access still uneven despite high mobile penetration  #Widowed and oldest-old more vulnerable to access barriers | #DISHA‑G is rare for India — offers integrated health, demographic, and environmental data for older populations in low-resource settings |
| 37 | Factors associated with public and private healthcare utilization for outpatient care among older adults in India: A Wagstaff’s decomposition of Andersen’s behavioral model | Margubur Rahaman et al., 2024 | India | Cross‑sectional, quantitative analysis | #To analyse determinants of outpatient public, private, and other facility utilisation among older adults.  #To measure wealth-based inequalities in healthcare choice and decompose contributions of various factors.  #To inform strategies for reducing inequities in access towards universal health coverage. | #Predisposing factors: Age, education, caste, region, residence type — significant for both public & private  #Enabling factors: Wealth quintile, current work status, insurance, distance strong influence  #Need factors: Purpose (consultation, check-up, injury), chronic disease, self‑rated health — important across facility types | Public sector provides low-cost care, benefiting poorer groups.  National/state insurance schemes exist (e.g., PM-JAY, RSBY).  Immunisation services strong in public facilities. | #Public sector underutilised due to poor infrastructure, staff shortages, quality concerns.  #Outpatient care often excluded from insurance schemes.  #Rural areas face facility shortages and long travel distances.  High OOPE in private sector widens inequities. | #Higher education improves awareness and decision-making on facility choice.  #Wealthier households can access quality care (public/private).  #Urban residence offers wider provider options. | #Poorer and less educated groups rely on lower-quality or costly services, risking financial distress.  #Rural-urban and caste disparities hinder equal access.  #Low health literacy leads to underuse of preventive services. | #Private sector preference is high even among low-income groups — potentially causing long-term poverty.  #Distance to facility influences public use but not private use (willingness to travel for quality). |
| 38 | Horizontal Equity of Outpatient Care Utilization in Elderly under Universal Coverage Scheme: Evidence from 2 Area Health Managements in Thailand | Intarates et. Al, 2025 | Thailand | Quantitative econometric analysis | #To quantify horizontal inequity in outpatient healthcare utilization (number of visits, expenses) among elderly with chronic NCDs in two Thai health regions  #To decompose need and non-need factors contributing to inequity | #All CI and HI values positive and significant → healthcare use (visits & costs) was pro-rich  #More educated (higher SES) elderly had more visits & incurred higher expenses  #Pro-rich pattern persisted even after controlling for health needs | UCS provides universal entitlement to outpatient services across regions  Equitable geographical distribution of tertiary and secondary hospitals in the studied provinces  Strong referral system for chronic disease management | #Persistent pro-rich bias despite universality — richer & more educated elderly used more and higher-cost services  #Limited outreach and engagement for lower-SES, less educated elderly  #Mobility issues and referral gaps for rural elderly  #Unequal resource concentration at tertiary level hospitals in certain provinces | #Higher education correlates with better awareness and care-seeking  #Some agricultural and service sector occupations linked with higher service use due to NCD burden  #Strong family structures in rural areas may support hospital visits | #Low education and low-SES groups making fewer visits despite high needs  #Informal/unstable employment → reduced affordability for indirect costs (travel, opportunity cost)  #Widowed and socially isolated elderly face greater access barriers | #Inequity appears more strongly associated with non-need SES factors than pure health needs |
| 39 | Understanding the association of disability with multimorbidity, and healthcare utilization in India’s older adult population: insights from cross-sectional evidence of SAGE‑2 | Ritik Agrawal et al., 2024 | India | Cross-sectional, nationally representative household survey | Descriptive statistics | #To estimate the prevalence of disability and multimorbidity in India’s older adults #To assess the association between disability and multimorbidity  #To analyse healthcare utilization patterns among older adults with disability, multimorbidity | Presence of public sector facilities and charitable providers available locally  #Existing national elderly care framework (National Programme for Health Care of the Elderly)  Availability of primary healthcare as first point of contact in rural areas | #Heavy reliance on private sector despite higher cost burden  Public sector lacks specialist services, accessibility, and infrastructure for geriatric care. Outpatient & long-term disability care under-covered in insurance schemes  #Gaps in integrated, home-based care for multimorbidity & disability | #Family caregiving still prevalent in rural India  #Literacy and higher wealth improve healthcare access and service choice  Married/partnered elderly tend to have better support for seeking care | #Poverty strongly associated with greater disability risk and poorer care access  Gender disparity — women more likely to have disability but face multi-dimensional access barriers  #Rural-urban gap in facility proximity and specialist availability  #Low health literacy, especially in low-SES elderly, limits preventive care uptake | #Multimorbidity and disability have a bi-directional relationship, potentially mediated by chronic inflammation and functional decline  #Private sector preference likely due to availability of specialised services, shorter wait times, and better amenities |
